# Trends on cancer incidence and mortality in Pasto, Colombia. 15 years experience

**DOI:** 10.25100/cm.v49i1.3616

**Published:** 2018-03-30

**Authors:** María Clara Yépez, Daniel Marcelo Jurado, Luisa Mercedes Bravo, Luis Eduardo Bravo

**Affiliations:** 1 Registro Poblacional de Cáncer del Municipio de Pasto, Grupo de investigación Salud Pública, Centro de Estudios en Salud (CESUN), Universidad de Nariño, Pasto, Colombia.; 2 Departamento de Patología, Universidad del Valle, Cali, Colombia.

**Keywords:** cancer, incidence, mortality, trends, Disease Prevention (DeCS), cáncer, incidencia, mortalidad, tendencias, Prevención de Enfermedades

## Abstract

**Introduction:**

In Colombia it is necessary to continue producing quality and continuously updated information on the magnitude of cancer, derived from population-based cancer registries to contribute to decision making, and implementation of strategies for health promotion, prevention and treatment of cancer in order to reduce the impact on the population.

**Objective:**

To describe the incidence, mortality and cancer trends in Pasto-Colombia from 1998 to 2012.

**Methods:**

Observational descriptive study of morbi - mortality due to malignant tumours in Pasto. The collection, processing and systematization of the data, was carried out according to international standards for population-based cancer registries. The incidence and mortality rates were calculated by period, sex, age and tumour site.

**Results:**

During the period 1998-2012 there were 8,010 new cases of cancer, of them, 57.7% occurred in females. There were 4,214 deaths reported, 52.0% in females. The incidence (*p* men= 0.7, *p* females= 0.3) and mortality (*p* males= 1.0, *p* females= 0.0) did not present significant changes over 15 years of observation and the tumours that cause greater morbi-mortality affect the stomach, cervix uteri, breast and prostate.

**Conclusions:**

Cancer in general, continues to be a serious health problem for the population of Pasto. The global behaviour of cancer incidence and mortality, identify the need to promote and strengthen promotion and prevention programs, especially focused on tumours of the stomach, prostate, breast and cervix uteri that produce greater morbidity and mortality in the population.

## Resumen

**Introducción:**

En Colombia es necesario que se continúe produciendo información de calidad y actualizada sobre la magnitud del cáncer a partir de datos de los registros poblacionales de cáncer para contribuir a la toma de decisiones e implementación de estrategias de promoción de la salud, prevención y tratamiento del cáncer con el objetivo de disminuir el impacto en la población.

**Objetivo:**

Describir la incidencia, mortalidad y tendencia del cáncer en Pasto-Colombia durante 1998-2012.

**Métodos:**

Estudio observacional descriptivo de la morbi-mortalidad por tumores malignos en Pasto. La recolección, procesamiento y sistematización de los datos se realizó de acuerdo a parámetros estandarizados internacionalmente para registros poblacionales de cáncer. Las tasas de incidencia y mortalidad se calcularon por periodo, sexo, edad y localización del tumor.

**Resultados:**

En el período 1998-2012 se registraron 8,010 casos nuevos de cáncer, de ellos, 57.7% se presentaron en mujeres. Se reportaron 4,214 muertes, 52.0% en mujeres. La incidencia (*p* hombres= 0.7; *p* mujeres= 0.3) y mortalidad (*p* hombres= 1.0; *p* mujeres= 0.0) no presentó cambios significativos durante 15 años de observación y los tumores que causan mayor morbi-mortalidad afectan al estómago, cuello uterino, mama y próstata.

**Conclusiones:**

El cáncer en general continúa siendo un importante problema de salud para la población de Pasto. El comportamiento global de la incidencia y la mortalidad por cáncer, evidencian la necesidad de fomentar y fortalecer programas de promoción y prevención, enfocados especialmente hacia los tumores de estómago, próstata, mama y cuello uterino que producen mayor morbi-mortalidad en la población.

## Introduction

In recent decades, cancer has become one of the leading causes of mortality worldwide. According to the World Health Organization (WHO), this disease represents 21% of total deaths due to non-communicable diseases, and it is the second cause of death after cardiovascular diseases (48%) ^1^. Different agencies both governmental and non-governmental organizations, have stressed the importance of understanding the impact of this disease, not only in terms of mortality but also of morbidity (incidence, prevalence and burden) and have promoted the creation and strengthening of epidemiological surveillance and information systems of a regional and national nature, called Population-based Cancer Registries (PBCR) ^2^. Thus, in 1966 the International Association of Cancer Registries (IACR) was founded, whose main objective is to promote the monitoring of cancer in populations through PBCR with internationally standardized methodological guidelines that allow the production of scientific evidence with quality criteria such as: *comparability, comprehensiveness, validity and timeliness* in order to base public policies and interventions for the prevention and control of cancer, as well as to evaluate its effectiveness ^3^ ^,^ ^4^.

According to estimations published by GLOBOCAN, an epidemiological surveillance system derived from PBCR, cancer is not only a problem exclusively of high-income countries(HIC), low and middle-income countries (LMIC) allow more than half of the annual cancer burden with 7 million new cases (56%) and 4.8 millions of deaths (64%), although they are the least prepared to face this situation. Without planning and control interventions in these populations, the burden of disease due to cancer will increase by 70%. Therefore, cancer is considered a threat to human and economic development in these countries. In Latin America and the Caribbean it is estimated that each year there are around 900,000 new cases, 542,000 deaths, and more than 2 million people living with cancer ^5^.

In Colombia, the National Cancer Institute (NCI) estimated that for the period 2007-2011 the morbi- mortality from cancer at national and departmental level from the mortality information in combination with data produced by five PBCR that followed the methods of the IACR and that have produced information regarding the magnitude, distribution and tendency of the malignant tumours in certain populations that correspond to 8.9% of the national population (Cali, Bucaramanga, Manizales, Barranquilla and Pasto) ^6^ ^,^ ^7^. For this period, Colombia estimated a total of 62,812 new cases of cancer per year, 29,734 cases in males and 33,084 cases in females were estimated for the country. In males, the highest incidence of tumours occurred in: prostate, stomach, colon and rectum. In females affect the: breast, cervix, uteri, colon and rectum. In the same period, there were 32,653 cancer deaths, 16,081 deaths in men and 16,572 deaths in females. The main causes of cancer mortality in men were tumours of: stomach, prostate and lung; in females the tumours of: breast, cervix uteri and stomach. According to this report, for the Department of Nariño, where is located Pasto, stomach cancer in both males and females produces the highest mortality rate ^8^.

The Cancer Registry of Pasto, processes information on cancer cases that occur in the rural and urban area of Pasto, Colombia, which according to the 2005 census has a population of 382,422 inhabitants, 47.8% males, 52.2% females. The population is spread out 81.7% in the urban area, and 18.3% in the rural area. Previous studies conducted over 1998-2007 showed that the cancer which produces the highest morbi-mortality in males was the stomach, and in females the cervix uteri.

In order to observe the behaviour of the different types of tumours, the characterization of morbi-mortality due to cancer in Pasto was carried out over the period 1998-2012, and the analysis of the trend of the incidence and mortality of the main types of tumours over a period of fifteen years. This study was done to contribute to decision-making and the implementation of strategies to promote health, prevention and treatment of cancer that help to mitigate the impact of this disease in the region ^9^ ^,^ ^10^.

## Materials and Methods

### Design and population

**Figure 1:**
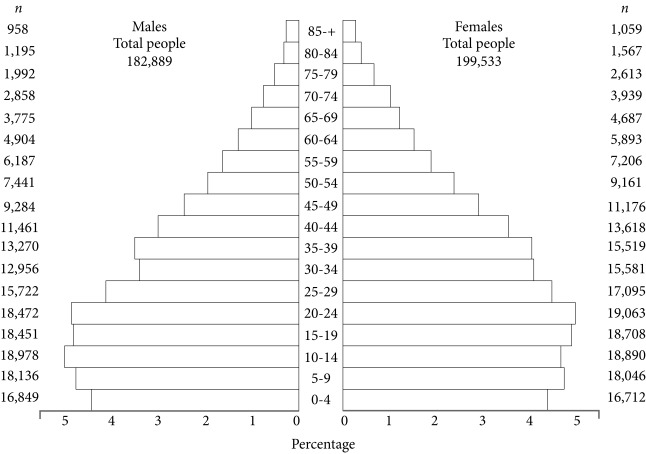
Average annual person-years by sex and age group. Colombia, Pasto 2005 Resource. General Census -2005. Colombian National Administrative Department of Statistics (DANE).

A descriptive observational study of all the malignant tumours and cancer deaths presented on residents of The Municipality of Pasto-Colombia during 1998-2012 was conducted. The city is located in south western Colombia and by the middle of the study period there were approximately 350,000 inhabitants (2005), of which 74.7% with health care and 57.0% living in low socioeconomic neighborhoods ^11^ ^,^ ^12^ (Fig. 1).

Ninety per cent of the tumours that were included were microscopically confirmed (histology, cytology, and bone marrow aspiration); the rest, were identified using other methods valid for the IACR (imaging, exploratory surgery, endoscopy, clinical and death certificate) in population-based studies. The percentage distribution of indices of data quality varies according to the primary site of the tumour and is shown in Table 1.

**Table 1:** Indices of data quality. Males and females of Pasto, Colombia, 1998-2012

Site	Males				Females				ICD-10 code		
	n	%MV	% DCO	%Others	MI	n	% DCO	%MV	%Others	MI	
Oral cavity	39	84.6	7.7	7,7	0.4	45	6.7	77.8	15.6	0.3	C00-14
Oesophagus	73	86.3	12.3	1.4	1.0	36	16.7	77.8	5.6	0.9	C15
Stomach	804	80.7	12.6	6.7	0.7	495	17.2	75.2	7.7	0.8	C16
Small intestine	13	69.2	15.4	15.4	0.3	12	8.3	83.3	8.3	0.5	C17
Colon and rectum	177	84.2	6.2	9.6	0.5	242	8.7	80.6	10.7	0.6	C18-20
Anus	5	100.0	0.0	0.0	0.5	19	0.0	100.0	0.0	0.3	C21
Liver	82	47.6	25.6	26.8	1.4	92	38.0	37.0	25.0	1.4	C22
Gallbladder	57	45.6	14.0	40.4	0.4	154	16.2	59.1	24.7	0.6	C23-24
Pancreas	78	26.9	23.1	50.0	0.9	116	25.0	30.2	44.8	1.1	C25
Nose, sinuses etc.	7	100.0	0.0	0.0	0.7	5	20.0	60.0	20.0	1.0	C30-31
Larynx	34	85.3	8.8	5.9	0.6	2	0.0	100.0	0.0	0.0	C32
Lung	168	60.1	20.8	19.0	1.1	117	23.1	61.5	15.4	1.2	C33-34
Other thoracic organs	7	100	0.0	0.0	0.3	7	0.0	100.0	0.0	0.5	C37-38
Bone	19	94.7	5.3	0.0	0.6	21	9.5	81.0	9.5	0.9	C40-41
Melanoma of skin	56	98.2	0.0	1.8	0.2	108	0.0	99.1	0.9	0.2	C43
Other Skin	33	93.9	6.1	0.0	0.3	35	8.6	88.6	2.9	0.6	C44
Mesothelioma	6	100.0	0.0	0.0	1.0	4	0.0	100.0	0.0	0.0	C45
Kaposi sarcoma	9	100.0	0.0	0.0	0.0	1	0.0	100.0	0.0	-	C46
Connective and soft tissue	66	95.5	0.0	4.5	0.3	41	0.0	97.6	2.4	0.4	C47,49
Breast	5	100	0.0	0.0	0.5	790	1.6	94.7	3.7	0.3	C50
Vulva						16	6.3	87.5	6.3	0.4	C51
Vagina						10	0.0	90.0	10.0	0.3	C52
Cervix uteri						733	4.8	91.8	3.4	0.4	C53
Corpus uteri						129	3.9	93.8	2.3	0.2	C54
Uterus unspecified						9	55.6	33.3	11.1	2.0	C55
Ovary						217	5.5	83.9	10.6	0.4	C56
Placenta						6	0.0	83.3	16.7	0.0	C58
Penis	36	91.7	2.8	5.6	0.3						C60
Prostate	626	82.3	8.3	9.4	0.3						C61
Testis	105	98.1	0.0	1.9	0.1						C62
Other male genital organs	3	100.0	0.0	0.0	0.0						C63
Kidney	45	71.1	8.9	20.0	0.2	41	7.3	85.4	7.3	0.2	C64
Renal Pelvis	1	100.0	0.0	0.0	0.0	2	0.0	100.0	0.0	0.0	C65
Bladder	92	88.0	6.5	5.4	0.3	52	5.8	82.7	11.5	0.5	C67
Other urinary organs	2	100.0	0.0	0.0	1.0	2	0.0	100.0	0.0	-	C68
Eye	17	100.0	0.0	0.0	0.1	19	0.0	100.0	0.0	0.0	C69
Brain, nervous system	106	78.3	6.6	15.1	0.6	99	17.2	66.7	16.2	0.7	C70-72
Thyroid	60	91.7	5.0	3,3	0,2	302	1.7	95.7	2.6	0.1	C73
Adrenal gland	1	0.0	100.0	0.0	-	1	0.0	0.0	100.0	-	C74
Other endocrine glands	1	0.0	100.0	0.0	-	2	0.0	100.0	0.0	0.0	C75
Lymphomas	259	96.5	1.2	2.3	0.4	210	1.0	97.1	1.9	0.4	C81-82,85,96
Multiple myeloma	39	89.7	0.0	10.3	0.6	42	4.8	83.3	11.9	0.7	C90
Leukemia	147	96.6	2.0	1,4	0,6	115	6.1	93.0	0.9	0.8	C90-95
Myelodysplastic syndromes	12	100.0	0.0	0.0	0.3	15	0.0	100.0	0.0	0.3	CIE-O-3: 998_/3
Myeloproliferative disorders	3	100.0	0.0	0.0	0.5	9	100.0	0.0		0.3	CIE-O3: 9950/3, 996_3/3, 9975/3
Other and unspecified	133	52.6	16.5	30.8	0.7	211	15.2	60.2	24.6	0.5	C26,39,48,76,80
All sites	3,423	80.6	9.3	10.1	0.6	4,584	8.5	83.0	8.5	0.5	C00-96

% MV: percentage of cases with microscopic verification (cytology-hematology and histology of the primary tumor)

% DCO: percentage of cases conducted on death certificate-only

% others: percentage of cases diagnosed by other methods (imaging, endoscopy and clinical)

MI: Mortality/Incidence

For mortality analysis all deaths recorded on the death certificate with basic cause of death C00-C99 according to ICD-10 (International Classification of Diseases 10th edition) were included. Deaths coded as "non-specific uterus" (C55), corresponding to 12% of deaths due to uterine cancer, were distributed between the category "cervix uteri" and "body of the uterus" taking into account the proportion of deaths observed by age, according to IACR guidelines^3^. 98.0% of the deaths were certified by medical personnel, the rest were certified by non-medical health personnel. The percentage of deaths with unknown age was 0.3%, and the percentage of deaths with unknown primary site (C76-C80) was 5.0%. Incident and mortality cases do not necessarily refer to the same person.

For each person with an incident tumour and cancer death recorded in the period, information was collected on demographic (age, sex) and clinical conditions (date of incidence or death, primary site of the tumour, cause of death). The date of incidence corresponds to the first chronological event of diagnostic confirmation of the disease or in case of lack of data the date of death was used.

The information was collected in an active, continuous and systematic way in all the health institutions that generate information on cancer: hospitals, clinics, oncology units, pathology and haematology laboratories, medical centers, specialized offices and the Municipal Health Secretariat, responsible for processing the death certificates. In addition, to guarantee the completeness of the data, databases of hospital discharges, Beneficiaries Selection System for Social Programs (SISBEN in Spanish), National Attorney General's Office, National Registry of Civil Status, Registry of patients of third level hospitals , the Solidarity and Guarantee Fund - FOSYGA and the mortality database of the National Administrative Department of Statistics (DANE in Spanish).

The cases were entered into the CanReg5 system for the elimination of duplicates, processing and complementation of data. The identification of primary multiple tumours follows the IACR standards ^13^. For the validation of the internal consistency between the variables, an automatic check was carried out with the IACRcrg Tools program version 2.05 and the rare cases were resolved in a scientific committee formed by specialists or by consulting with the Cali Cancer Registry.

### Analysis of the information

For the analysis of incidence and mortality, frequencies were calculated such as absolute, relative, crude, specific rates (by period, sex, age and site of the tumour or cause of death according to the ICD-10, grouped into large categories.) and standardized by age (ASR) to the world population standard (SEGI) by the direct method ^7^ ^,^ ^14^. The DANE population estimates and projections by the middle of the period were used as a denominator at risk for calculating the rates, which were calculated considering the basic components of the population dynamics: fertility, mortality, and migration (internal and international) from the population base determined in the 2005 census, and adjusted by conciliation of the General Census (census 1985, 1993 and 2005) ^15^. Cases without age (0.15%), basal cell carcinoma and squamous cell of the skin were excluded.

The incidence and mortality results are presented in specific Tables or Charts and the analysis of the main sites was intensified because they are diseases of great relevance for the region. Crude and standardized incidence and mortality rates are expressed per 100,000 males-year or females-year.

To assess the trend of incidence and mortality, a global analysis between five-year periods was made and the percentage change in rates between the last two periods was estimated. Additionally, a trend analysis of annual incidence and mortality rates was performed using a segmented linear regression or joinpoint, accepting a maximum of three change points (joinpoints) with four linear segments respectively. The annual percentage of change (APC) was estimated in each possible segment generated between each point and the average annual percentage change (AAPC) was calculated for the entire period. All possible models were adjusted with the weighted least squares method and the model selection was made with the Montecarlo permutations test. All analyses were carried out in the SEER stat and Joinpoint 4.0 program produced by the Surveillance Research Program of the National Cancer Institute of the United States ^16^.

### Ethical considerations

This study is classified as without risk research according to resolution 8430 of 1993 of the Ministry of Health of Colombia, since the information comes from secondary sources and has no direct contact or intervention of the biological, physiological, psychological or social variables of the individuals studied. The handling of information follows the confidentiality rules established by the International Agency for Cancer Research (IACR) that regulate the use of data for scientific purposes without the disclosure of personal data, guaranteeing respect and non-maleficence towards patients. On the other hand, the Cancer Registry of Pasto and the investigations that derive from it have agreements with the sources of information to guarantee the adequate flow of the data.

## Results

### Global incidence

During the period 1998-2012 in the municipality of Pasto, 8,010 cases of cancer were identified. (ASR: 145.1 cases per 100,000 persons-year), 3,426 in males (ASR: 139.1 cases per 100,000 males-year) and 4,584 cases in females (ASR: 150.3 cases) per 100,000 females-year) (Tables 2 and 3).

**Table 2:** Cancer incidence rates by tumour site, crude and age- standardized per 100,000 males-year. Pasto, Colombia, 1998-2002, 2003-2007 and 2008-2012

Site	1998-2002				2003-2007				2008-2012				PC (%) 2003-2007 and 2008-2012		ICD-10 code
	n	%	CR	ASR	n	%	CR	ASR	n	%	CR	ASR	CR	ASR	
Oral cavity	15	1.5	1.8	2.3	13	1.1	1.4	1.7	11	0.9	1.1	1.1	-21.4	-35.3	C00-14
Oesophagus	35	3.6	4.1	4.6	23	2	2.5	3	15	1.2	1.5	1.5	-40.0	-50.0	C15
Stomach	296	30.1	34.8	42.6	258	22	28.2	32.6	250	19.7	25.3	26.7	-10.3	-18.1	C16
Small intestine	4	0.4	0.5	0.6	5	0.4	0.5	0.6	4	0.3	0.4	0.5	-20.0	-16.7	C17
Colon and rectum	42	4.3	4.9	5.9	57	4.9	6.2	7.3	78	6.1	7.9	8.4	27.4	15.1	C18-20
Anus	1	0.1	0.1	0.1	2	0.2	0.2	0.3	2	0.2	0.2	0.2	0.0	-33.3	C21
Liver	26	2.6	3.1	3.7	27	2.3	3	3.5	29	2.3	2.9	3.0	-3.3	-14.3	C22
Gallbladder	17	1.7	2	2.6	21	1.8	2.3	2.8	19	1.5	1.9	2.2	-17.4	-21.4	C23-24
Pancreas	18	1.8	2.1	2.6	36	3.1	3.9	4.4	24	1.9	2.4	2.8	-38.5	-36.4	C25
Nose, sinuses etc.	1	0.1	0.1	0.2	2	0.2	0.2	0.3	4	0.3	0.4	0.5	100.0	66.7	C30-31
Larynx	6	0.6	0.7	0.9	16	1.4	1.7	2.3	12	0.9	1.2	1.4	-29.4	-39.1	C32
Trachea, bronchus and lung	47	4.8	5.5	6.5	60	5.1	6.6	7.5	61	4.8	6.2	7.0	-6.1	-6.7	C33-34
Other thoracic organs	0	0.0	0.0	0.0	4	0.3	0.4	0.5	3	0.2	0.3	0.3	-25.0	-40.0	C37-38
Bone	8	0.8	0.9	0.9	6	0.5	0.7	0.8	5	0.4	0.5	0.5	-28.6	-37.5	C40-41
Melanoma of skin	11	1.1	1.3	1.4	16	1.4	1.7	2.1	29	2.3	2.9	3.1	70.6	47.6	C43
Other Skin	7	0.7	0.8	0.9	13	1.1	1.4	1.8	13	1.0	1.3	1.5	-7.1	-16.7	C44
Mesothelioma	1	0.1	0.1	0.2	2	0.2	0.2	0.2	3	0.2	0.3	0.3	50.0	50.0	C45
Kaposi sarcoma	0	0.0	0.0	0.0	1	0.1	0.1	0.1	8	0.6	0.8	0.8	700.0	700.0	C46
Connective and soft tissue	14	1.4	1.6	1.7	32	2.7	3.5	3.9	20	1.6	2	2.3	-42.9	-41.0	C47,49
Breast	0	0.0	0.0	0.0	2	0.2	0.2	0.2	3	0.2	0.3	0.4	50.0	100.0	C50
Penis	7	0.7	0.8	1.1	11	0.9	1.2	1.4	18	1.4	1.8	1.9	50.0	35.7	C60
Prostate	163	16.6	19.1	23.2	213	18.1	23.3	27.3	250	19.7	25.3	27.3	8.6	0.0	C61
Testis	21	2.1	2.5	2.3	43	3.7	4.7	4.3	41	3.2	4.1	3.6	-12.8	-16.3	C62
Other Male genital organs	1	0.1	0.1	0.2	1	0.1	0.1	0.1	1	0.1	0.1	0.1	0.0	0.0	C63
Kidney	15	1.5	1.8	2.1	15	1.3	1.6	1.7	15	1.2	1.5	1.8	-6.3	5.9	C64
Renal pelvis	0	0.0	0.0	0.0	0	0.0	0.0	0.0	1	0.1	0.1	0.1			C65
Bladder	35	3.6	4.1	4.8	28	2.4	3.1	3.7	29	2.3	2.9	3.0	-6.5	-18.9	C67
Other urinary organs	0	0.0	0.0	0.0	1	0.1	0.1	0.1	1	0.1	0.1	0.1	0.0	0.0	C68
Eye	8	0.8	0.9	1.1	4	0.3	0.4	0.6	5	0.4	0.5	0.6	25.0	0.0	C69
Brain, nervous system	26	2.6	3.1	3.2	45	3.8	4.9	5.2	35	2.8	3.5	3.7	-28.6	-28.8	C70-72
Thyroid	12	1.2	1.4	1.5	20	1.7	2.2	2.7	28	2.2	2.8	2.6	27.3	-3.7	C73
Adrenal gland	0	0.0	0.0	0.0	1	0.1	0.1	0.1	0	0.0	0.0	0.0	-100.0	-100.0	C74
Other endocrine glands	0	0.0	0.0	0.0	1	0.1	0.1	0.1	0	0.0	0.0	0.0	-100.0	-100.0	C75
Lymphomas	72	7.3	8.5	9	87	7.4	9.5	10.9	100	7.9	10.1	10.6	6.3	-2.8	C81-82,85,96
Multiple myeloma	8	0.8	0.9	1	8	0.7	0.9	1	23	1.8	2.3	2.5	155.6	150.0	C90
Leukemia	40	4.1	4.7	4.5	52	4.4	5.7	6.1	55	4.3	5.6	5.9	-1.8	-3.3	C90-95
Myelodysplastic syndromes	0	0.0	0.0	0.0	3	0.3	0.3	0.4	9	0.7	0.9	0.8	200.0	100.0	CIE-O-3: 998_/3
Myeloproliferative disorders	0	0.0	0.0	0.0	0	0.0	0.0	0.0	3	0.2	0.5	0.5			CIE-O3: 9950/3, 996_3/3, 9975/3
Other and unspecified	25	2.5	2.9	3.3	45	3.8	4.9	5.4	63	5.0	6.4	6.8	30.6	25.9	C26,39,48,76,80
All sites	982	100.0	115.3	135.0	1,174	100.0	128.3	147.1	1,270	100.0	128.6	136.4	0.2	-7.3	C00-96
All sites except C44	975	99.3	114.5	134.1	1,161	98.9	126.9	145.4	1,257	99.0	127.3	134.9	0.3	-7.2	C00-43,45-96

CR: Crude rate of incidence x 100,000 males-year;

ASR: Age-standardized rates (SEGI world population standard) x 100,000 males-year;

**Table 3:** Cancer incidence rates by tumour site, crude and age-standardized per 100,000 females-year. Pasto, Colombia, 1998-2002, 2003-2007 and 2008-2012

Site	1998-2002				2003-2007				2008-2012				PC (%) 2003-2007 and 2008-2012		ICD-10 code
	n	%	CR	ASR	n	%	CR	ASR	n	%	CR	ASR	CR	ASR	
Oral cavity	17	1.3	1.9	1.9	21	1.4	2.1	2.1	7	0.4	0.7	0.6	-66.7	-71.4	C00-14
Oesophagus	12	0.9	1.3	1.3	13	0.8	1.3	1.2	11	0.7	1.0	1	-23.1	-16.7	C15
Stomach	183	13.5	20.2	20.2	175	11.4	17.6	17.1	137	8.1	12.8	11.8	-27.3	-31.0	C16
Small intestine	3	0.2	0.3	0.3	3	0.2	0.3	0.3	6	0.4	0.6	0.6	100.0	100.0	C17
Colon and rectum	63	4.6	7.0	6.8	75	4.9	7.5	7.5	104	6.2	9.7	9	29.3	20.0	C18-20
Anus	5	0.4	0.6	0.6	8	0.5	0.8	0.8	6	0.4	0.6	0.5	-25.0	-37.5	C21
Liver	26	1.9	2.9	2.7	32	2.1	3.2	3.0	34	2.0	3.2	2.8	0.0	-6.7	C22
Gallbladder	59	4.3	6.5	6.4	48	3.1	4.8	5.0	47	2.8	4.4	4	-8.3	-20.0	C23-24
Pancreas	21	1.5	2.3	2.3	54	3.5	5.4	5.5	41	2.4	3.8	3.7	-29.6	-32.7	C25
Nose, sinuses etc.	0	0	0.0	0.0	3	0.2	0.3	0.3	2	0.1	0.2	0.1	-33.3	-66.7	C30-31
Lung	0	0	0.0	0.0	1	0.1	0.1	0.1	1	0.1	0.1	0.1	0.0	0.0	C32
Trachea, bronchus and lung	23	1.7	2.5	2.3	37	2.4	3.7	3.8	57	3.4	5.3	4.8	43.2	26.3	C33-34
Other thoracic organs	2	0.1	0.2	0.3	2	0.1	0.2	0.1	3	0.2	0.3	0.3	50.0	200.0	C37-38
Bone	5	0.4	0.6	0.6	8	0.5	0.8	0.7	8	0.5	0.7	0.7	-12.5	0.0	C40-41
Skin melanoma	31	2.3	3.4	3.3	32	2.1	3.2	3.2	45	2.7	4.2	4	31.3	25.0	C43
Other skin	10	0.7	1.1	1.1	10	0.6	1.0	1.1	15	0.9	1.4	1.2	40.0	9.1	C44
Mesothelioma	3	0.2	0.3	0.4	0	0.0	0.0	0	1	0.1	0.1	0.1			C45
Kaposi sarcoma	0	0.0	0.0	0.0	1	0.1	0.1	0.1	0	0.0	0.0	0.0	-100.0	-100.0	C46
Connective and soft tissue	15	1.1	1.7	1.6	15	1.0	1.5	1.6	11	0.7	1.0	0.9	-33.3	-43.8	C47,49
Breast	219	16.1	24.2	24.4	258	16.7	25.9	27.1	313	18.6	29.3	27.7	13.1	2.2	C50
Vulva	5	0.4	0.6	0.5	5	0.3	0.5	0.6	6	0.4	0.6	0.5	20.0	-16.7	C51
Vagina	7	0.5	0.8	0.7	0	0.0	0.0	0.0	3	0.2	0.3	0.3			C52
Cervix uteri	251	18.5	27.7	27.0	272	17.7	27.3	27.4	210	12.5	19.6	18.0	-28.2	-34.3	C53
Corpus uteri	39	2.9	4.3	4.6	45	2.9	4.5	4.7	45	2.7	4.2	4.3	-6.7	-8.5	C54
Uterus unspecified	4	0.3	0.4	0.4	1	0.1	0.1	0.1	4	0.2	0.4	0.4	300.0	300.0	C55
Ovary	63	4.6	7.0	7.1	70	4.5	7.0	7.1	84	5.0	7.9	7.3			C56
Placenta	2	0.1	0.2	0.2	4	0.3	0.4	0.3	0	0.0	0.0	0	-100.0	-100.0	C58
Kidney	13	1	1.4	1.4	13	0.8	1.3	1.4	15	0.9	1.4	1.5	7.7	7.1	C64
Renal pelvis	1	0.1	0.1	0.1	0	0.0	0.0	0.0	1	0.1	0.1	0.1			C65
Bladder	18	1.3	2.0	2.0	14	0.9	1.4	1.5	20	1.2	1.9	1.7	35.7	13.3	C67
Other urinary organs	0	0.0	0.0	0.0	1	0.1	0.1	0.1	1	0.1	0.1	0.1	0.0	0.0	C68
Eye	10	0.7	1.1	1.2	7	0.5	0.7	0.7	2	0.1	0.2	0.2	-71.4	-71.4	C69
Brain, nervous system	23	1.7	2.5	2.7	27	1.8	2.7	2.8	49	2.9	4.6	4.4	70.4	57.1	C70-72
Thyroid	71	5.2	7.8	7.7	92	6.0	9.2	9	139	8.3	13.0	11.8	41.3	31.1	C73
Adrenal gland	1	0.1	0.1	0.2	0	0.0	0.0	0.0	0	0.0	0.0	0.0			C74
Other endocrine glands	0	0.0	0.0	0.0	0	0.0	0.0	0.0	2	0.1	0.2	0.2			C75
Lymphomas	64	4.7	7.1	6.6	71	4.6	7.1	7.3	75	4.5	7.0	6.3	-1.4	-13.7	C81-82,85,96
Multiple myeloma	7	0.5	0.8	0.8	19	1.2	1.9	2.1	16	1.0	1.5	1.4			C90
Leukemia	35	2.6	3.9	4.2	35	2.3	3.5	3.7	45	2.7	4.2	4.1	20.0	10.8	C90-95
Myelodysplastic syndromes	0	0.0	0.0	0.0	3	0.2	0.3	0.3	12	0.7	1.1	0.9	266.7	200.0	CIE-O-3: 998_/3
Myeloproliferative disorders	0	0.0	0.0	0.0	1	0.1	0.1	0.1	8	0.5	0.9	0.8	800.0	700.0	CIE-O3: 9950/3, 996_3/3, 9975/3
Other and unspecified	48	3.5	5.3	5.6	65	4.2	6.5	6.4	98	5.8	9.2	8.8	41.5	37.5	C26,39,48,76,80
All sites	1359	100.0	149.9	149.6	1541	100	154.7	156.1	1684	100	157.7	147.1	1.9	-5.8	C00-96
All sites except C44	1,349	99.3	148.8	148.5	1,531	99.4	153.7	155.1	1669	99.1	156.3	145.9	1.7	-5.9	C00-43,45-96

CR: Crude rate of incidence x 100,000 males-year;

ASR: Age-standardized rates (SEGI world population standard) x 100,000 males-year;

By sex and period, in males there were in 1998-2002, 982 cases (ASR: 135 cases per 100,000 males-year), for 2003-2007 1,174 cases (ASR: 147.1 cases per 100,000 males-year) and in 2008-2012 1,270 cases (ASR: 136.4 cases per 100,000 males-year). In females during 1998-2002 there were 1,359 cases (ASR: 149.6 cases per 100,000 females-year), for 2003-2007: 1,541 cases (ASR: 156.1 cases per 100,000 females-year) and in 2008-2012: 1,684 cases (ASR: 147.1 cases per 100,000 females-year). The percentage of change in the global incidence rates between the last two periods was 0.2 in males and in females of 1.0%

**Figure 2:**
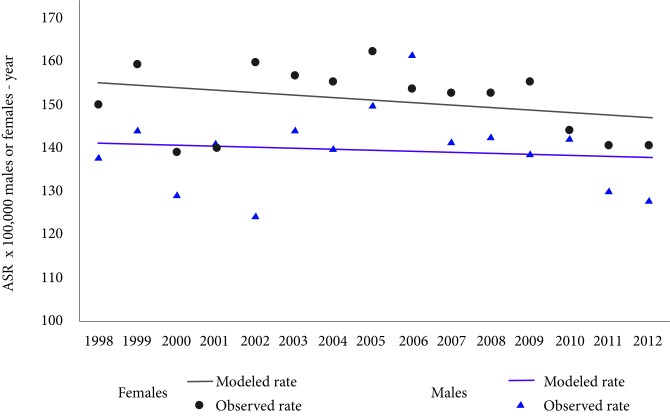
Trend of age- standardized global rates of cancer incidence. Males and females of Pasto, Colombia, 1998-2012. APC: Annual Percentage of change. ASR: Age-standardized rates (SEGI world population standard) x 100,000 males or females-year

The incidence trend indicates that there were no significant changes in the incidence rates in both males and females during the analysed period (*p*-value males= 0.7, *p*-value females= 0.3), that means the incidence was stable. The average annual incidence rates standardized by age were 139.7 cases per 100,000 males-year and 150.7 cases per 100,000 females-year (Fig. 2).

### Incidence by age

**Figure 3:**
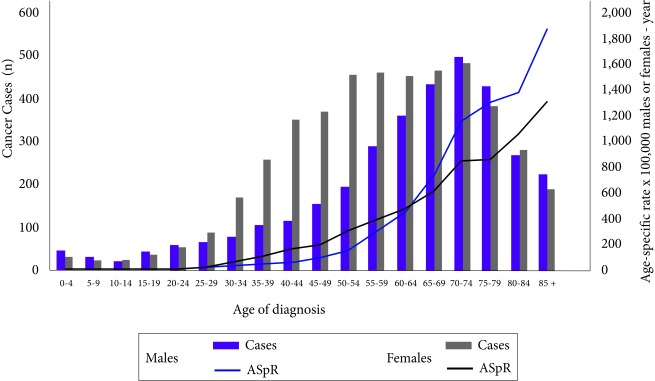
Cases and specific rates by age per 100,000 males or females-year of incidence of cancer, Pasto, Colombia, 1998-2012. ASpR: Age-Specific rate x 100,000 males or females-year

Cases and cancer incidence rates increase by age; 55% of incident cases in males and 40% in females occurred after 65 years, while 2% of cases in both genders occurred in children under 15 years of age (Fig. 3). The average age of diagnosis in males was 62.3 years (Standard deviation SD= 18.7 years) and in females of 58.2 years (SD= 18.1 years).

### Incidence by tumour site

The most frequent tumours over the 15 years in males were tumours of the: Stomach (23.5%), prostate (18.3%), lymphomas (7.6%) colon and rectum (5.2%) and lung (4.9%). In females were: breast (17.3%), cervix uteri (16.0%), stomach (10.8%), thyroid (6.6%) and colon and rectum (5.3%). This behaviour was observed when analysing each of the three five-year periods that comprise the study period (Tables 2 and 3).

**Figure 4:**
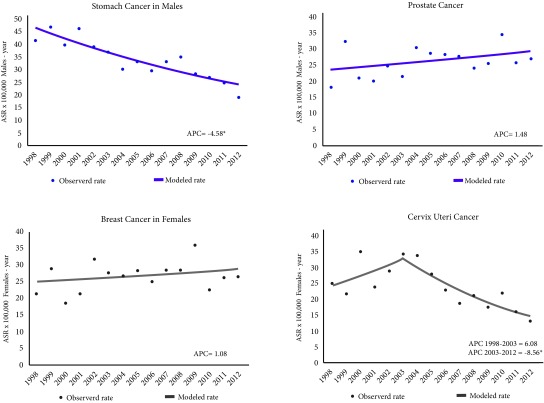
Trend of age-standardized incidence rates for the most frequent tumours. Pasto, Colombia, males and females 1998-2012. APC = Annual percentage of change. ASR: Age-standardized rates (SEGI world population standard) x 100,000 males-year. * Statistically significant (p <0.005)

The incidence of stomach tumours in males decreased significantly by 4.6% (*p*-value = 0.0) annually from an ASR of 41.5 in 1998 to 19.1 cases per 100,000 males-year in 2012. In females, the incidence of cervix uteri decreased 8.6% (*p*= 0.0) annually from 2003 from an ASR of 34.2 to 13.4 cases per 100,000 females-year in 2012. The incidence of prostate tumours (*p*= 0.2) and breast (*p*= 0.3) was constant and the average of its annual incidence rates standardized by age was 26 cases per 100,000 males-year and 26.4 cases per 100,000 females-year, respectively (Fig. 4).

### Global mortality 

Over the period 1998-2012 in the municipality of Pasto there were 4,221 deaths due to cancer (ASR: 75.9 deaths per 100,000 people-year), 1,949 in males (ASR: 78 deaths per 100,000 males-year) and 2,272 deaths in females (ASR: 74.4 deaths per 100,000 females-year (Tables 4 and 5).

**Table 4:** Cancer mortality rates by tumour site, crude and age-standardized per 100,000 males-year. Pasto, Colombia, 1998-2002, 2003-2007 and 2008-2012.

Site	1998-2002				2003-2007				2008-2012				PC (%) 2003-2007 and 2008-2012		>ICD-10 code
	n	%	CR	ASR	n	n	%	CR	ASR	n	n	%	CR	ASR	
Oral cavity	3	0.5	0.4	0.4	4	0.6	0.4	0.4	9	1.2	0.9	0.9	125.0	125.0	C00-14
Oesophagus	22	4.0	2.6	3.2	25	3.9	2.7	3.1	25	3.3	2.5	2.6	-7.4	-16.1	C15
Stomach	190	34.4	22.3	26.6	172	26.5	18.8	21.5	200	26.7	20.2	21.5	7.4	0.0	C16
Small intestine	0	0.0	0.0	0.0	2	0.3	0.2	0.2	2	0.3	0.2	0.2	0.0	0.0	C17
Colon and rectum	15	2.7	1.8	1.9	34	5.2	3.7	4.2	50	6.7	5.1	5.3	37.8	26.2	C18-20
Liver	28	5.1	3.3	4.3	48	7.4	5.2	6.4	31	4.1	3.1	3.2	-40.4	-50.0	C22
Pancreas	20	3.6	2.3	3.1	28	4.3	3.1	3.4	27	3.6	2.7	3.0	-12.9	-11.8	C25
Lung	62	11.2	7.3	9.2	61	9.4	6.7	7.3	66	8.8	6.7	7.3	0.0	0.0	C34
Skin melanoma and Other skin	9	1.6	1.1	1.3	2	0.3	0.2	0.3	11	1.5	1.1	1.1	450.0	266.7	C43-44
Breast	2	0.4	0.2	0.2	1	0.2	0.1	0.1	0	0.0	0.0	0.0	-100.0	-100.0	C50
Próstate	66	11.9	7.7	9.4	81	12.5	8.9	9.2	92	12.3	9.3	9.0	4.5	-2.2	C61
Bladder	9	1.6	1.1	1.4	7	1.1	0.8	0.7	17	2.3	1.7	1.7	112.5	142.9	C67
Lymphomas and myelomas	37	6.7	4.3	4.4	45	6.9	4.9	5.7	55	7.4	5.6	6.0	14.3	5.3	C90 ,C81-82,85,96
Leucemias	25	4.5	2.9	2.8	21	3.2	2.3	2.4	42	5.6	4.2	4.3	82.6	79.2	C90-95
Other malignant tumours	65	11.8	7.6	8.8	117	18.1	12.8	14.4	121	16.2	12.2	12.9	-4.7	-10.4	
All sites	553	100.0	64.9	76.9	648	100.0	70.8	79.4	748	100.0	75.6	79.0	6.8	-0.5	C00-96

CR: Crude rate of mortality x 100,000 males-year;

ASR: Age-standardized rates (SEGI world population standard) x 100,000 males-year;

**Table 5:** Cancer mortality rates by tumour site, crude and age-standardized per 100,000 females-year. Pasto, Colombia, 1998-2002, 2003-2007 and 2008-2012.

Site	1998-2002				2003-2007				2008-2012				PC (%) 2003-2007 and 2008-2012		ICD-10 code
	n	%	CR	ASR	n	%	CR	ASR	n	%	CR	ASR	CR	ASR	
Oral cavity	2	0.3	0.2	0.2	3	0.4	0.3	0.3	7	0.7	0.7	0.6	133.3	100.0	C00-14
Oesophagus	8	1.3	0.9	1.0	11	1.6	1.1	1.0	13	1.3	1.2	1.1	9.1	10.0	C15
Stomach	131	21.8	14.5	14.1	121	17.3	12.1	12.0	128	13.2	12.0	10.9	-0.8	-9.2	C16
Small intestine	3	0.5	0.3	0.3	3	0.4	0.3	0.3	1	0.1	0.1	0.1	-66.7	-66.7	C17
Colon and rectum	25	4.2	2.8	2.5	41	5.8	4.1	4.1	74	7.6	6.9	6.3	68.3	53.7	C18-20
Liver	31	5.2	3.4	3.6	44	6.3	4.4	4.2	46	4.7	4.3	4.1	-2.3	-2.4	C22
Pancreas	36	6.0	4.0	4.0	52	7.4	5.2	5.3	43	4.4	4.0	3.7	-23.1	-30.2	C25
Lung	38	6.3	4.2	4.0	41	5.8	4.1	4.2	62	6.4	5.8	5.5	41.5	31.0	C34
Skin melanoma and Other skin	13	2.2	1.4	1.4	11	1.6	1.1	1.0	17	1.8	1.6	1.4	45.5	40.0	C43-44
Breast	69	11.5	7.6	7.6	73	10.4	7.3	7.6	115	11.9	10.8	10.4	47.9	36.8	C50
Cervix uteri	107	17.8	11.8	11.6	65	9.3	6.5	6.6	111	11.4	10.4	9.5	60.0	43.9	C53
Corpus uteri	2	0.3	0.2	0.2	14	2.0	1.4	1.5	5	0.5	0.5	0.5	-64.3	-66.7	C54
Ovary	0	0.0	0.0	0.0	30	4.3	3.0	3.2	56	5.8	5.2	5.0	73.3	56.3	C56
Bladder	7	1.2	0.8	0.8	5	0.7	0.5	0.5	14	1.4	1.3	1.3	160.0	160.0	C67
Lymphomas and myelomas	34	5.7	3.8	3.8	34	4.9	3.4	3.5	48	4.9	4.5	4.2	32.4	20.0	C90 ,C81-82,85,96
Leukemias	30	5.0	3.3	3.3	24	3.4	2.4	2.5	41	4.2	3.8	3.5	58.3	40.0	C90-95
Other malignant tumours	65	10.8	7.2	7.2	129	18.4	13.0	13.2	189	19.5	17.7	16.3	36.2	23.5	
All sites	601	100.0	66.3	65.6	701	100.0	70.4	71.2	970	100.0	90.7	84.3	28.8	18.4	C00-96

CR: Crude rate of mortality x 100,000 females-year;

ASR: Age- standardized rates (SEGI world population standard) x 100,000 females-year;

According to sex and period, over 1998-2002 period, 553 deaths occurred in males (ASR: 76.9 deaths per 100,000 males-year), for 2003-2007, 648 deaths (ASR 79.4 deaths per 100,000 males-year) and in 2008-2012, 748 deaths (ASR: 79 per 100,000 males-year deaths). Over 1998-2002 there were 601 deaths in females (ASR: 65.6 deaths per 100,000 females-year), for 2003-2007, 701 deaths (ASR: 71.2 deaths per 100,000 females-year) and in 2008-2012, 970 deaths (ASR: 84.3 deaths per 100,000 females-year).

**Figure 5:**
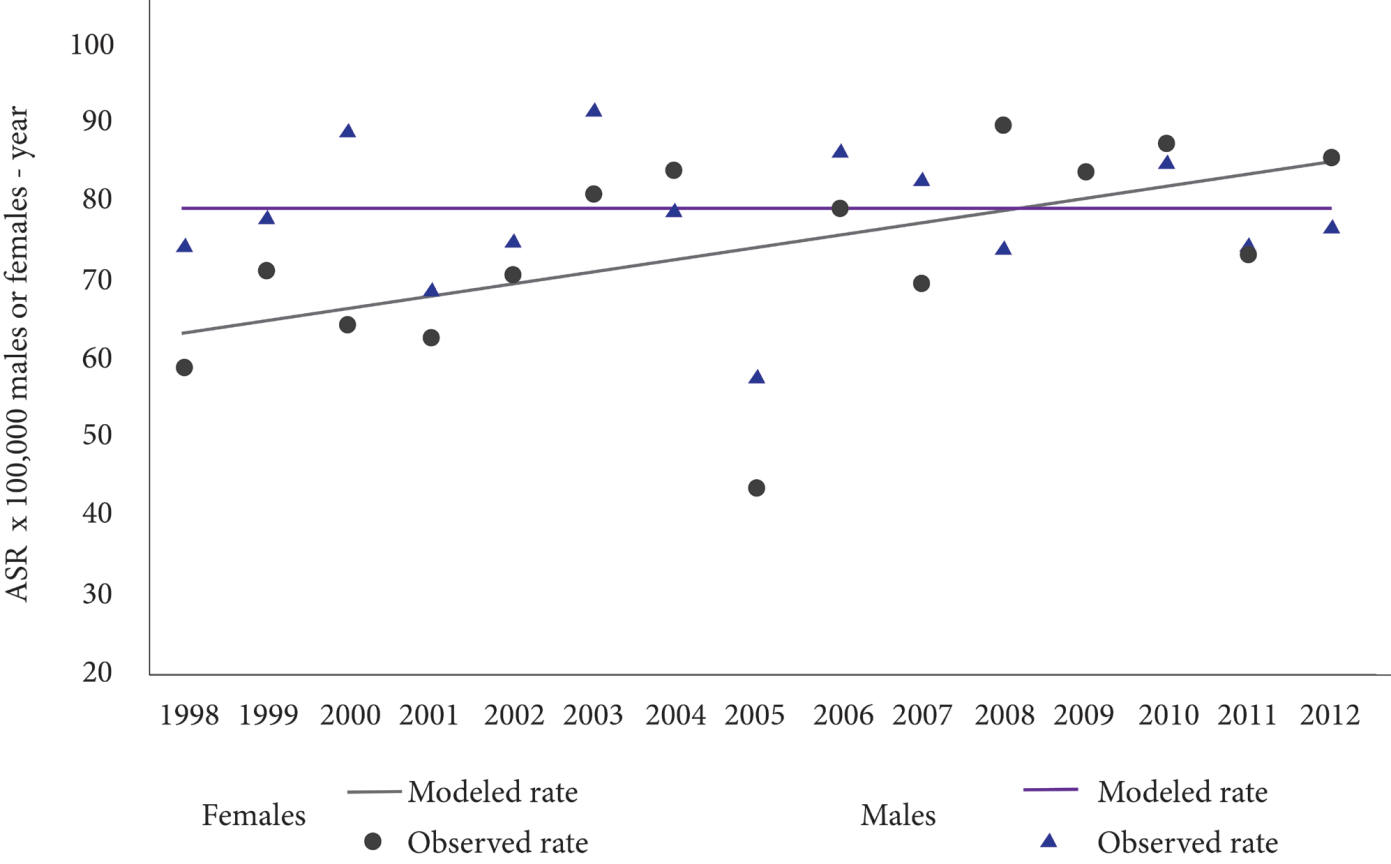
Trend of age-standardized global rates of cancer mortality. Males and females of Pasto, Colombia, 1998-2012. APC: Annual percentage of change. ASR: Age- standardized rates (SEGI world population standard) x 100,000 males-year. * Statistically significant (p <0.005)

The trend in mortality indicates that there are no significant changes in mortality rates in males (*p*-value males = 1.0, *p*-value females = 0.0), the average annual mortality rate standardized by age was 78.5 deaths per 100,000 males-year. While in females mortality rates reached 2.1% per year from an ASR: of 58.6 to 86.0 deaths per 100,000 females-year, in 1998 and 2012 respectively (Fig. 5). The average in the three quinquennial periods of the ratio between the mortality-incidence rates (M: I) was 56 deaths per 100 diagnostic cases in males and 49 deaths per 100 diagnostic cases in females.

**Figure 6:**
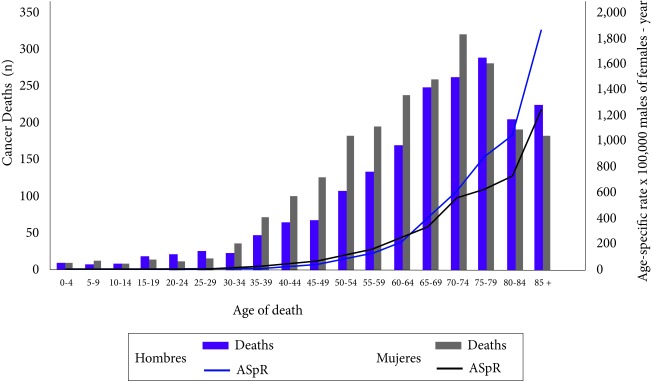
Deaths and death age-specific rates due to cancer per 100,000 males or females-year of Pasto, Colombia, 1998-2012. ASR: Age-Specific rate x 100,000 Males or females-year

Like the incidence, mortality showed the highest percentage of cases after 65 years old, both in males (63.9%) and in females (55.2%) (Fig. 6). The average age of death for males was estimated at 66 years (SD: 17.8 years) and for females at 63.7 years (SD: 17.1).

### Mortality by tumour site

Over 1998-2012, the main causes of cancer mortality in males were tumours of: Stomach (28.8%), prostate (12.3%) lung (9.7%), lymphomas and myelomas (7%) and liver (5.5%). In females were tumours of: Stomach (16.7%), cervix uteri (12.5%), breast (11.3%), lung (6.2%), colon and rectum (6.2%). This behaviour was observed during the three five-year periods that comprise the period of study (Tables 4 and 5).

**Figure 7:**
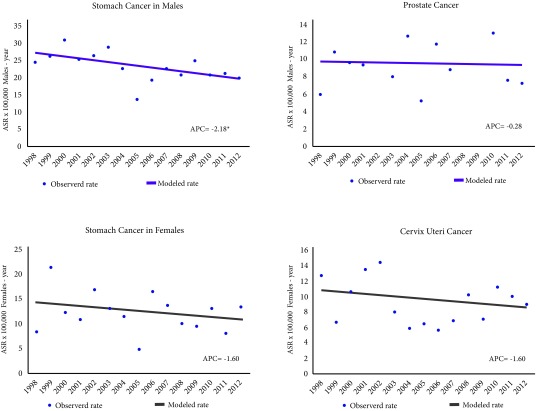
Trend of age-standardized mortality rates for the most frequent tumours. Pasto, Colombia, males and females 1998-2012. APC: Annual percentage of change. ASR: Age-standardized rates (SEGI world population standard) x 100,000 males-year. * Statistically significant (*p*-value <0.005).

Mortality caused by stomach tumours in males decreased significantly (*p*-value = 0.0) 2.18% annually from an ASR of 24.5 in 1998 to 19.9 deaths per 100,000 males-year in 2012. In females, breast cancer mortality increased (*p*-value= 0.0) 3% annually from an ASR of 6.3 in 1998 to 10.8 deaths per 100,000 females-year in 2012. Mortality from prostate tumours (*p*-value= 0.9), stomach tumours in females (*p*-value= 0.4) and cervix uteri (*p*-value= 0.4) was constant and the average of their annual mortality rates standardized by age was 9.3 deaths per 100,000 males-year, 12.3 deaths per 100,000 females-years and 9.2 deaths per 100,000 females-year, respectively (Fig. 7). 

## Discussion

The population characterization on cancer incidence and mortality carried out by this study is part of the initiative to implement programs for its control promoted by the Organization of the United Nations, the World Health Organization (WHO), the specialized agency in cancer IARC and at the national level the Ministry of Health and Social Protection and the National Institute of Cancerology of Colombia ^10^ ^,^ ^17^. These organizations consider that the implementation of any program to control this disease and its outcomes should be supported by scientific evidence and defined as: *"approaches from public health, designed to contribute to the reduction of cancer morbi-mortality, improve the patients´quality of life with this condition through the systematic and equitable implementation of strategies of prevention, early diagnosis, treatment and palliation based on evidence, for the optimal use of available resources; prioritizing in those vulnerable populations with greater cancer burden and intervening in those tumours that produce higher incidence and mortality*" ^3^ ^,^ ^18^ ^-^ ^20^.

At the regional level, thanks to the advances of the Cali Cancer Registry for the systematic reporting of data on incidence, mortality and survival to cancer, the Union for International Cancer Control (UICC), selected the city of Cali as one of the first cities in the world to implement the C/Can 2025 initiative: Challenge of cities against cancer, that consists of involving all the stakeholders of each city in the design, planning and implementation of solutions for cancer care. The data on the characterization of cancer morbi- mortality provided by the Cali Cancer Registry for five decades will be the only source of evidence for evaluating the effects of the implementation of this strategy ^21^. Following the model of Cali, it is intended that the results presented in this study constitute the baseline about the cancer situation in the municipality of Pasto to assess the effects of the implementation of *the national program for cancer control* that Colombia addressed in 2010 ^9^.

In general, it is considered that the global incidence rate for the Municipality of Pasto (ASR: 139.1 cases per 100,000 males-year and ASR: 150.3 cases per 100,000 females-year) and in other geographically close regions are low; Cali-Colombia (ASR: 205 cases per 100,000 males-year and ASR: 186 cases per 100,000 females-year), Manizales-Colombia (ASR: 156 cases per 100,000 males-year and ASR: 165 cases per 100,000 females-year), Bucaramanga-Colombia (ASR: 154 cases per 100,000 males-year and ASR: 157 cases per 100,000 females-year), Quito-Ecuador (ASR: 193 cases per 100,000 males-year and ASR: 199 cases per 100,000 females-year), Manabí-Ecuador (ASR: 89 cases per 100,000 males-year and ASR: 102 cases per 100,000 females-year) ^24^. The results observed are similar in other Latin American countries and contrary to those reported by the majority of North American and oceanic registers ^24^.

The behaviour of the global incidence rates of the populations can be associated to lifestyles, diagnostic capacity in the health system, but mainly to the demographic and epidemiological transition; in populations of North America, Oceania, Europe and Asia with a significant population aging, the risk of chronic diseases increases, especially those of late presentation such as cancer, in contrast to those populations with a younger population structure such as in Latin America and Africa, which have greater public health problems related to communicable diseases ^25^ ^,^ ^26^.

When contrasting incidence rates with mortality rates, to establish the mortality-incidence ratio (M: I) it can be observed that, although the majority of North American registries have higher rates of incidence in both males and females, the mortality and incidence ratio reaches the lowest values (M:I= 30-40), which means that for every 100 cases that are diagnosed there are between 30 and 40 cancer deaths in the same period. On the other hand, in Latin American countries the ratio M:I range between 60 and 80, its mean, for every 100 incident cases there are around 80 deaths. This is an indirect indicator of the quality of the health system in relation to diagnostic tests for the identification of new cancer cases and oncological treatment services to avoid deaths ^7^.

When comparing the incidence and mortality trends reported by other registries that have published at least 15 years consecutively in IC5, it is observed that the incidence of stomach tumours decreased significantly in most populations, primarily in those from European countries where the annual percentage of change (APC) reported ranges between -5.1% and -3.1%, with less decrease in North American, Oceanic and some European countries, the APC varies between -2.9% and -2.3% and with the lowest decrease in Latin American populations with an APC that ranges between -2.2% and -0.9%. Only the registry of kyadondo county-Uganda and Goiania-Brazil, have reported an increase in the incidence of stomach cancer where the APC is 2.1% and 0.1% respectively. In the Municipality of Pasto, the trend of incidence and mortality from stomach cancer has achieved a significant decrease, this behaviour is explained by the study on the trend of incidence and mortality from stomach cancer in Cali, which indicates that it is probably related to the decrease of the prevalent rates of infection by Helicobacter pylori, the improvement of life habits and early detection in the population, however, for the case of the Municipality of Pasto, it is suggested to study in depth the causes of this behaviour behaviour ^27^ ^,^ ^28^.

The trend of the incidence of prostate tumours in the world has increased significantly in most populations, in an accelerated way in Latin American populations, some European, Asian and oceanic with an APC that varies between 5.2% and 11.0%, has slightly increased in some European, Asian and Oceanic countries with an APC that fluctuates between 3.4% and 3.9%, and in Africa, North America and some European countries has increased very little, with an APC ranging between 3.3% and 1.5%. Only Indian populations (3 records) reported that the incidence trend of prostate cancer has decreased slightly with an APC of 0.2%. For the Municipality of Pasto, incidence and mortality rates over time have remained constant without showing a statistically significant change. The analysis of this behavior should be studied to establish the factors related to early diagnosis and treatment.

In females, the trend in the incidence of breast cancer is increasing globally and is accentuated in populations of African and some European and Asian registries with an APC ranging between 5.3% and 2.2%, in Latin American, oceanic and some European populations increased moderately with an APC that varies between 2.1% and 1.3%, and very little in North American populations and some Asians with an APC that ranges between 0.3% and 1.2%. This behaviour is probably caused by the increase in obesity, physical inactivity, and changes in reproductive and other behavioural habits ^29^. The tendency of the mortality by breast cancer varies between regions: populations of European and North American countries show decreasing tendencies, contrary to the presented in the populations of South American countries. In Pasto the incidence rate remains constant and mortality has a tendency to increase similar to other populations of South American countries ^30^.

The decrease in the incidence rates of cervical cancer worldwide is very evident, with a greater decrease in Latin American, Oceanic and some European populations with an APC that ranges between -2.7% and -8.6%, an average decrease in populations in North America, some European and Asian with an APC that fluctuates between -1.4% and -2.6% and with a reduced decrease in some European and Asian populations with an APC that oscillates between -0.3% and -1.1%, behaviour contrary to that reported by African populations where the incidence trend increased with an APC of 3.9%. In Pasto the tendency of the incidence of cervical cancer has lowered nevertheless the tendency of the mortality stays constant, results that reflect difficulties in the early detection and opportune treatment.

For the Municipality of Pasto the analysis of the incidence, mortality and behaviour of the tendency of the types of cancer of greater occurrence becomes a base for the evaluation of the impact of the measures of prevention, treatment, implementation of new technologies and investigations that promote actions to control the impact of the disease on the population.
