# Supplementary material for: Trends on cancer incidence and mortality in Pasto, Colombia. 15 years experience
Source: Colomb Med (Cali). 2018 Mar 30;49(1):42–54. doi: 10.25100/cm.v49i1.3616 (PMC6018824; doi:10.25100/cm.v49i1.3616)
Supplement: Related texts

# Research article: Tendencia de la incidencia y mortalidad por cáncer en Pasto, Colombia; 15 años de experiencia

## Introducción

En las últimas décadas el cáncer se ha convertido en una de las principales causas de mortalidad a nivel mundial. Según cifras de la Organización Mundial de la Salud (OMS), esta enfermedad representa el 21% del total de las muertes por enfermedades no trasmisibles y es la segunda causa de muerte después de las enfermedades cardiovasculares (48%) ^1^. Diferentes agencias y organizaciones gubernamentales y no gubernamentales han recalcado la importancia de conocer el impacto de esta enfermedad no solo en términos de mortalidad sino también de morbilidad (incidencia, prevalencia y carga) y han impulsado la creación y fortalecimiento de sistemas de información y vigilancia epidemiológica de carácter regional o nacional, llamados Registros Poblacionales de Cáncer (RPC) ^2^. Es así que en 1966 se fundó la Asociación Internacional de Registros de Cáncer (IACR), cuyo objetivo principal es promover el monitoreo del cáncer en las poblaciones mediante RPCs con lineamientos metodológicos estandarizados internacionalmente que permitan producir evidencia científica con criterios de calidad como: *comparabilidad*, *exhaustividad, validez y oportunidad* con la finalidad de fundamentar políticas públicas e intervenciones para la prevención y control del cáncer, así como también evaluar su efectividad ^3^^,^^4^. 

Según estimaciones publicadas por GLOBOCAN, un sistema de vigilancia epidemiológica derivada de los RPCs, el cáncer no es un problema propio de los países de ingresos altos (PIA), los países de ingresos medios y bajos (PIMB) soportan más de la mitad de la carga anual por cáncer con 7 millones de casos nuevos (56%) y 4.8 millones de muertes (64%), aunque son los menos preparados para afrontar esta situación. Sin intervenciones de planificación y control en estas poblaciones, la carga de la enfermedad debido al cáncer aumentará un 70%. Por ello, se considera que el cáncer supone una amenaza al desarrollo humano y económico en estos países. En América Latina y el Caribe se estima que cada año existen alrededor de 900,000 nuevos casos, 542,000 muertes, y más de 2 millones de personas que conviven con la enfermedad ^5^. 

En Colombia, el Instituto Nacional de Cancerología E.S.E, estimó para el periodo 2007-2011 la morbi-mortalidad por cáncer a nivel nacional y departamental a partir de la información de mortalidad en combinación con cifras producidas por cinco registros poblacionales de cáncer que siguen la metodología de la IACR y que han producido información respecto a la magnitud, distribución y tendencia de los tumores malignos en determinadas poblaciones que corresponden al 8.9% de la población nacional (Cali, Bucaramanga, Manizales, Barranquilla y Pasto) eps^6^^,^^7^. Para este periodo se estimaron en Colombia, un total de 62,812 casos nuevos anuales de cáncer, 29,734 casos en hombres y 33,084 casos en mujeres. En hombres, la mayor incidencia de tumores se presentó en: próstata, estómago y colon y recto. En mujeres: mama, cuello del útero, y colon y recto. En el mismo periodo se registraron 32,653 muertes por cáncer, 16,081 en hombres y 16,572 en mujeres. Las principales causas de mortalidad por cáncer en hombres fueron los tumores de: estómago, próstata y pulmón; en mujeres los tumores de: mama, cuello del útero y estómago. Según este informe, para el Departamento de Nariño, donde se ubica Pasto, el cáncer de estómago tanto en hombres como en mujeres produce la tasa más alta de mortalidad eps^8^. 

El registro Poblacional de cáncer del Municipio de Pasto procesa información de los casos de cáncer que ocurren en el área rural y urbana del municipio de Pasto-Colombia que según el censo de 2005 tiene una población de 382,422 habitantes, 47.8% hombres 52.2% mujeres. La población se distribuye el 81.7% en la zona urbana y 18.3% en la zona rural. Los estudios realizados en el período 1998-2007 mostraron que el cáncer que produce mayor morbi mortalidad en hombres fue el de estómago y en mujeres el de cuello uterino.

Con el propósito de observar el comportamiento de los diferentes tipos de tumores, se realizó la caracterización de la morbi mortalidad por cáncer en el municipio de Pasto durante el período 1998-2012 y el análisis de la tendencia de la incidencia y mortalidad de los principales tipos de tumores en un período de quince años, con el fin de contribuir a la toma de decisiones y a la implementación de estrategias de promoción de la salud, prevención y tratamiento del cáncer que contribuyan a mitigar el impacto de la enfermedad en la región ^9^^,^^10^.

## Materiales y Métodos

### Diseño y población de estudio

Se realizó un estudio observacional descriptivo de todos los tumores malignos y las muertes por cáncer presentadas durante 1998-2012 en personas residentes en el Municipio de Pasto-Colombia. El municipio está localizado en el suroccidente de Colombia y para la mitad del periodo de estudio contaba con aproximadamente 350,000 habitantes (2005), de los cuales el 74.7% con aseguramiento en salud y 57% viviendo en estratos socioeconómicos bajos. ^11^^,^^12^ (Fig. 1). 

**Figura 1:** Promedio anual persona-año por sexo y grupo de edad. Colombia, Pasto 2005 Fuente. Censo general -2005. Departamento Administrativo Nacional de Estadística de Colombia (DANE).

El 90% de los tumores que se incluyeron fueron confirmados histopatológicamente (histología, citología, y aspirado de médula ósea); el resto, mediante otros métodos de diagnóstico válidos según la IARC (imagenología, cirugía exploratoria, endoscopia, clínico y por certificado de defunción) para estudios de base poblacional. La distribución porcentual varía según la localización primaria del tumor y se presenta en la Tabla 1. 

**Tabla 1:** Índices de calidad de los datos según localización. Hombres y mujeres de Pasto, Colombia, 1998-2012

Localización	Hombres				Mujeres						Codigo CIE-10
	n	%VM	% SCD	%Otros	MI	n	% SCD	%VM	%Otros	MI	
Cavidad bucal	39	84.6	7.7	7.7	0.4	45	6.7	77.8	15.6	0.3	C00-14
Esófago	73	86.3	12.3	1.4	1.0	36	16.7	77.8	5.6	0.9	C15
Estómago	804	80.7	12.6	6.7	0.7	495	17.2	75.2	7.7	0.8	C16
Intestino delgado	13	69.2	15.4	15.4	0.3	12	8.3	83.3	8.3	0.5	C17
Colon y recto	177	84.2	6.2	9.6	0.5	242	8.7	80.6	10.7	0.6	C18-20
Ano	5	100.0	0.0	0.0	0.5	19	0.0	100.0	0.0	0.3	C21
Hígado	82	47.6	25.6	26.8	1.4	92	38.0	37.0	25.0	1.4	C22
Vesícula biliar	57	45.6	14.0	40.4	0.4	154	16.2	59.1	24.7	0.6	C23-24
Páncreas	78	26.9	23.1	50.0	0.9	116	25.0	30.2	44.8	1.1	C25
Fosas nasales y oído medio	7	100.0	0.0	0.0	0.7	5	20.0	60.0	20.0	1.0	C30-31
Laringe	34	85.3	8.8	5.9	0.6	2	0.0	100.0	0.0	0.0	C32
Pulmón	168	60.1	20.8	19.0	1.1	117	23.1	61.5	15.4	1.2	C33-34
Otros órganos torácicos	7	100.0	0.0	0.0	0.3	7	0.0	100.0	0.0	0.5	C37-38
Hueso	19	94.7	5.3	0.0	0.6	21	9.5	81.0	9.5	0.9	C40-41
Melanoma de piel	56	98.2	0.0	1.8	0.2	108	0.0	99.1	0.9	0.2	C43
Otros de piel	33	93.9	6.1	0.0	0.3	35	8.6	88.6	2.9	0.6	C44
Mesotelioma	6	100.0	0.0	0.0	1.0	4	0.0	100.0	0.0	0.0	C45
Sarcoma de Kaposi	9	100.0	0.0	0.0	0.0	1	0.0	100.0	0.0	-	C46
Tejidos conjuntivos y blandos	66	95.5	0.0	4.5	0.3	41	0.0	97.6	2.4	0.4	C47,49
Mama	5	100.0	0.0	0.0	0.5	790	1.6	94.7	3.7	0.3	C50
Vulva						16	6.3	87.5	6.3	0.4	C51
Vagina						10	0.0	90.0	10.0	0.3	C52
Cuello del útero						733	4.8	91.8	3.4	0.4	C53
Cuerpo del útero						129	3.9	93.8	2.3	0.2	C54
Útero NOS						9	55.6	33.3	11.1	2.0	C55
Ovario						217	5.5	83.9	10.6	0.4	C56
Placenta						6	0.0	83.3	16.7	0.0	C58
Pene	36	91.7	2.8	5.6	0.3						C60
Próstata	626	82.3	8.3	9.4	0.3						C61
Testículo	105	98.1	0.0	1.9	0.1						C62
Otros órganos genitales masculinos	3	100.0	0.0	0.0	0.0						C63
Riñón	45	71.1	8.9	20.0	0.2	41	7.3	85.4	7.3	0.2	C64
Pelvis renal	1	100.0	0.0	0.0	0.0	2	0.0	100.0	0.0	0.0	C65
Vejiga	92	88.0	6.5	5.4	0.3	52	5.8	82.7	11.5	0.5	C67
Otros órganos urinarios	2	100.0	0.0	0.0	1.0	2	0.0	100.0	0.0	-	C68
Ojo	17	100.0	0.0	0.0	0.1	19	0.0	100.0	0.0	0.0	C69
Cerebro y SNC	106	78.3	6.6	15.1	0.6	99	17.2	66.7	16.2	0.7	C70-72
Glándula tiroides	60	91.7	5.0	3.3	0.2	302	1.7	95.7	2.6	0.1	C73
Glándula suprarrenal	1	0.0	100.0	0.0	-	1	0.0	0.0	100.0	-	C74
Otras glándulas endocrinas	1	0.0	100.0	0.0	-	2	0.0	100.0	0.0	0.0	C75
Linfomas	259	96.5	1.2	2.3	0.4	210	1.0	97.1	1.9	0.4	C81-82,85,96
Mieloma múltiple	39	89.7	0.0	10.3	0.6	42	4.8	83.3	11.9	0.7	C90
Leucemias	147	96.6	2.0	1.4	0.6	115	6.1	93.0	0.9	0.8	C90-95
Síndromes mielodisplásicos	12	100.0	0.0	0.0	0.3	15	0.0	100.0	0.0	0.3	CIE-O-3: 998_/3
Desórdenes mieloproliferativos	3	100.0	0.0	0.0	0.5	9	100.0	0.0		0.3	CIE-O3: 9950/3, 996_3/3, 9975/3
Sitios NOS	133	52.6	16.5	30.8	0.7	211	15.2	60.2	24.6	0.5	C26,39,48,76,80
Todos los sitios	3,423	80.6	9.3	10.1	0.6	4,584	8.5	83.0	8.5	0.5	C00-96

% VM: porcentaje de casos con verificación microscópica (citología-hematología e histología del tumor primario)

% SCD: porcentaje de casos registrados sólo por certificados de defunción

% Otros: porcentaje de casos diagnosticados por otros métodos (Imagenología, endoscopia y clínicos)

MI: Mortalidad/Incidencia

Para el análisis de mortalidad se incluyeron las muertes por cáncer registradas en el certificado de defunción con causa básica de defunción C00-C99 según la CIE-10 (Clasificación Internacional de Enfermedades 10 edición). Las muertes codificadas como “útero inespecífico” (C55), que corresponden a un 12% de las muertes por cáncer uterino, fueron distribuidas entre la categoría “cuello del útero” y “cuerpo del útero” teniendo en cuenta la proporción de muertes observadas por edad, según guías de la IARC ^3^. El 98.0% de las muertes fueron certificadas por personal médico, el resto fue certificado por personal de salud no médico. El porcentaje de muertes con edad desconocida fue 0.3%, y el porcentaje de muertes con sitio primario desconocido (C76-C80) fue 5.0%. Los casos incidentes y de mortalidad no necesariamente hacen referencia a la misma persona.

De cada tumor incidente o muerte por cáncer registrada en el periodo se recolectó información sobre condiciones demográficas de la persona (edad, sexo) y clínicas (fecha de incidencia o muerte, localización primaria del tumor, causa de muerte). La fecha de incidencia corresponde al primer evento cronológico de confirmación diagnóstica de la enfermedad o en caso de carencia de datos se utilizó la fecha de defunción. 

La información se recolectó de forma activa, continua y sistemática en todas las instituciones de salud que generan información sobre cáncer: hospitales, clínicas, unidades de oncología, laboratorios de patología y hematología, centros médicos, consultorios especializados y la Secretaría de Salud Municipal, organismo encargado de procesar los certificados de defunción del municipio. Adicionalmente para garantizar al máximo la exhaustividad de los datos, se consultaron bases de datos de egresos hospitalarios, Sistema de Selección de Beneficiarios para programas Sociales (SISBEN), Procuraduría General de la Nación, Registraduría Nacional del Estado Civil, Registro de pacientes de hospitales de tercer nivel, el Fondo de Solidaridad y Garantía - FOSYGA y la base de mortalidad del Departamento Administrativo Nacional de Estadística (DANE).

Los casos se ingresaron al sistema CanReg5 para la eliminación de duplicados, procesamiento y complementación de datos. La identificación de tumores múltiples primarios sigue las normas de la IARC ^13^. Para la validación de la consistencia interna entre las variables se realizó un chequeo automático con el programa IARCcrg Tools versión 2.05 y los casos raros se resolvieron en el comité científico conformado por especialistas o en consulta al Registro Poblacional de Cáncer de Cali.

### Análisis de la información

Para el análisis de la incidencia y mortalidad se calcularon frecuencias absolutas, relativas, tasas crudas, específicas, periodo, sexo, edad y localización del tumor o causa de muerte según la CIE-10 (Clasificación Internacional de Enfermedades 10 edición), agrupados en grandes categorías.) y ajustadas por edad a la población mundial estándar (Segi) mediante el método directo ^7^^,^^14^. Como denominador a riesgo para el cálculo de las tasas se utilizaron las estimaciones y proyecciones poblacionales a mitad de año del DANE, calculadas considerando los componentes básicos de la dinámica poblacional: fecundidad, mortalidad, y migración (interna e internacional) a partir de la población base determinada en el censo de 2005 y ajustada mediante conciliación del Censo General (censo 1985, 1993 y 2005) ^15^. Se excluyeron los casos sin edad (0.15%), los carcinomas basocelulares y escamocelulares de piel. 

Los resultados de incidencia y mortalidad se presentan en Tablas o Figuras específicas y se profundizó el análisis de las principales localizaciones por tratarse de enfermedades de gran relevancia para la región. Las tasas de incidencia y mortalidad crudas y ajustadas se expresan por cada 100,000 hombres-año o mujeres-año.

Para evaluar la tendencia de la incidencia y mortalidad se realizó un análisis entre periodos quinquenales y se estimó el porcentaje de cambio de las tasas entre los dos últimos periodos. Adicionalmente se realiza un análisis de tendencia de las tasas de incidencia y mortalidad anuales utilizando una regresión lineal segmentada o joinpoint, aceptando como máximo 3 puntos de cambio (joinpoints) con cuatro segmentos lineales respectivamente. El porcentaje anual de cambio (PAC) fue estimado en cada posible segmento generado entre cada punto y el promedio del porcentaje anual de cambio (AAPC en inglés) fue calculado para todo el periodo. Todos los posibles modelos se ajustaron con el método de mínimos cuadrados ponderados y la selección del modelo se realizó con la prueba de permutaciones de Montecarlo. Todos los análisis se llevaron a cabo en el programa SEER stat y Joinpoint 4.0 producidos por el Surveillance Research Program of the National Cancer Institute de los Estados Unidos ^16^.

### Consideraciones éticas 

Este estudio se clasificó como investigación sin riesgo según la resolución 8430 de 1993 del Ministerio de Salud de Colombia, puesto que la información proviene de fuentes secundarias y no se tuvo un contacto directo o realizó intervenciones de las variables biológicas, fisiológicas, sicológicas o sociales de los individuos de estudio. El manejo de la información siguió las normas de confidencialidad establecidas por la Agencia Internacional de Investigación en Cáncer (IARC) que regulan el uso de los datos con fines científicos sin la divulgación de datos personales, garantizando el respeto y la no maleficencia hacia los pacientes. Por otra parte, el Registro Poblacional de Cáncer del Municipio de Pasto y las investigaciones que de él se derivan tienen convenios con las fuentes de información para garantizar el flujo adecuado de los datos.

## Resultados

### Incidencia global

Durante el periodo 1998-2012 en el municipio de Pasto se identificaron 8,010 casos de cáncer (TAE 145.1 casos por 100,000 personas-año), 3,426 en hombres (TAE 139.1 casos por 100,000 hombres-año) y 4,584 casos en mujeres (TAE 150.3 casos por 100,000 mujeres-año) (Tablas 2 y 3). 

**Tabla 2:** Tasas de incidencia de cáncer por localización, crudas y estandarizadas por edad por 100,000 hombres-año. Pasto, Colombia, 1998-2002, 2003-2007 y 2008-2012.

Localización	1998-2002				2003-2007				2008-2012				PC(%) 2003-2007 y 2008-2012		>Código CIE-10
	n	%	TC	TAE	n	%	TC	TAE	n	%	TC	TAE	CT	TAE	
Cavidad bucal	15	1.5	1.8	2.3	13	1.1	1.4	1.7	11	0.9	1.1	1.1	-21.4	-35.3	C00-14
Esófago	35	3.6	4.1	4.6	23	2.0	2.5	3.0	15	1.2	1.5	1.5	-40.0	-50.0	C15
Estómago	296	30.1	34.8	42.6	258	22.0	28.2	32.6	250	19.7	25.3	26.7	-10.3	-18.1	C16
Intestino delgado	4	0.4	0.5	0.6	5	0.4	0.5	0.6	4	0.3	0.4	0.5	-20.0	-16.7	C17
Colon y recto	42	4.3	4.9	5.9	57	4.9	6.2	7.3	78	6.1	7.9	8.4	27.4	15.1	C18-20
Ano	1	0.1	0.1	0.1	2	0.2	0.2	0.3	2	0.2	0.2	0.2	0.0	-33.3	C21
Hígado	26	2.6	3.1	3.7	27	2.3	3.0	3.5	29	2.3	2.9	3.0	-3.3	-14.3	C22
Vesícula biliar	17	1.7	2.0	2.6	21	1.8	2.3	2.8	19	1.5	1.9	2.2	-17.4	-21.4	C23-24
Páncreas	18	1.8	2.1	2.6	36	3.1	3.9	4.4	24	1.9	2.4	2.8	-38.5	-36.4	C25
Fosas nasales y oído medio	1	0.1	0.1	0.2	2	0.2	0.2	0.3	4	0.3	0.4	0.5	100.0	66.7	C30-31
Laringe	6	0.6	0.7	0.9	16	1.4	1.7	2.3	12	0.9	1.2	1.4	-29.4	-39.1	C32
Pulmón	47	4.8	5.5	6.5	60	5.1	6.6	7.5	61	4.8	6.2	7.0	-6.1	-6.7	C33-34
Otros órganos torácicos	0	0.0	0.0	0.0	4	0.3	0.4	0.5	3	0.2	0.3	0.3	-25.0	-40.0	C37-38
Hueso	8	0.8	0.9	0.9	6	0.5	0.7	0.8	5	0.4	0.5	0.5	-28.6	-37.5	C40-41
Melanoma de piel	11	1.1	1.3	1.4	16	1.4	1.7	2.1	29	2.3	2.9	3.1	70.6	47.6	C43
Otros de piel	7	0.7	0.8	0.9	13	1.1	1.4	1.8	13	1.0	1.3	1.5	-7.1	-16.7	C44
Mesotelioma	1	0.1	0.1	0.2	2	0.2	0.2	0.2	3	0.2	0.3	0.3	50.0	50.0	C45
Sarcoma de Kaposi	0	0.0	0.0	0.0	1	0.1	0.1	0.1	8	0.6	0.8	0.8	700.0	700.0	C46
Tejidos conjuntivos y blandos	14	1.4	1.6	1.7	32	2.7	3.5	3.9	20	1.6	2.0	2.3	-42.9	-41.0	C47,49
Mama	0	0.0	0.0	0.0	2	0.2	0.2	0.2	3	0.2	0.3	0.4	50.0	100.0	C50
Pene	7	0.7	0.8	1.1	11	0.9	1.2	1.4	18	1.4	1.8	1.9	50.0	35.7	C60
Próstata	163	16.6	19.1	23.2	213	18.1	23.3	27.3	250	19.7	25.3	27.3	8.6	0.0	C61
Testículo	21	2.1	2.5	2.3	43	3.7	4.7	4.3	41	3.2	4.1	3.6	-12.8	-16.3	C62
Otros órganos genitales masculinos	1	0.1	0.1	0.2	1	0.1	0.1	0.1	1	0.1	0.1	0.1	0.0	0.0	C63
Riñón	15	1.5	1.8	2.1	15	1.3	1.6	1.7	15	1.2	1.5	1.8	-6.3	5.9	C64
Pelvis renal	0	0.0	0.0	0.0	0	0.0	0.0	0.0	1	0.1	0.1	0.1			C65
Vejiga	35	3.6	4.1	4.8	28	2.4	3.1	3.7	29	2.3	2.9	3.0	-6.5	-18.9	C67
Otros órganos urinarios	0	0.0	0.0	0.0	1	0.1	0.1	0.1	1	0.1	0.1	0.1	0.0	0.0	C68
Ojo	8	0.8	0.9	1.1	4	0.3	0.4	0.6	5	0.4	0.5	0.6	25.0	0.0	C69
Cerebro y SNC	26	2.6	3.1	3.2	45	3.8	4.9	5.2	35	2.8	3.5	3.7	-28.6	-28.8	C70-72
Glándula tiroides	12	1.2	1.4	1.5	20	1.7	2.2	2.7	28	2.2	2.8	2.6	27.3	-3.7	C73
Glándula suprarrenal	0	0.0	0.0	0.0	1	0.1	0.1	0.1	0	0.0	0.0	0.0	-100.0	-100.0	C74
Otras glándulas endocrinas	0	0.0	0.0	0.0	1	0.1	0.1	0.1	0	0.0	0.0	0.0	-100.0	-100.0	C75
Linfomas	72	7.3	8.5	9.0	87	7.4	9.5	10.9	100	7.9	10.1	10.6	6.3	-2.8	C81-82,85,96
Mieloma múltiple	8	0.8	0.9	1.0	8	0.7	0.9	1.0	23	1.8	2.3	2.5	155.6	150.0	C90
Leucemias	40	4.1	4.7	4.5	52	4.4	5.7	6.1	55	4.3	5.6	5.9	-1.8	-3.3	C90-95
Síndromes mielodisplásicos	0	0.0	0.0	0.0	3	0.3	0.3	0.4	9	0.7	0.9	0.8	200.0	100.0	CIE-O-3: 998_/3
Desórdenes mieloproliferativos	0	0.0	0.0	0.0	0	0.0	0.0	0.0	3	0.2	0.5	0.5			CIE-O3: 9950/3, 996_3/3, 9975/3
Sitios NOS	25	2.5	2.9	3.3	45	3.8	4.9	5.4	63	5.0	6.4	6.8	30.6	25.9	C26,39,48,76,80
Todas las localizaciones	982	100.0	115.3	135.0	1,174	100.0	128.3	147.1	1,270	100.0	128.6	136.4	0.2	-7.3	C00-96
Todas las localizaciones sin piel	975	99.3	114.5	134.1	1,161	98.9	126.9	145.4	1,257	99.0	127.3	134.9	0.3	-7.2	C00-43,45-96

TC: Tasa cruda de incidencia x 100,000 hombres-año;

TAE: Tasa ajustada por edad x 100,000 hombres-año (población mundial estándar Segi);

PC: Porcentaje de cambio

**Tabla 3:** Tasas de incidencia de cáncer por localización, crudas y estandarizadas por edad por 100,000 mujeres-año. Pasto, Colombia, 1998-2002, 2003-2007 y 2008-2012.

Localización	1998-2002				2003-2007				2008-2012				PC(%) 2003-2007 y 2008-2012		>Código CIE-10
	n	%	TC	TAE	n	%	TC	TAE	n	%	TC	TAE	TC	TAE	
Cavidad bucal	17	1.3	1.9	1.9	21	1.4	2.1	2.1	7	0.4	0.7	0.6	-66.7	-71.4	C00-14
Esófago	12	0.9	1.3	1.3	13	0.8	1.3	1.2	11	0.7	1.0	1.0	-23.1	-16.7	C15
Estómago	183	13.5	20.2	20.2	175	11.4	17.6	17.1	137	8.1	12.8	11.8	-27.3	-31.0	C16
Intestino delgado	3	0.2	0.3	0.3	3.0	0.2	0.3	0.3	6	0.4	0.6	0.6	100.0	100.0	C17
Colon y recto	63	4.6	7.0	6.8	75	4.9	7.5	7.5	104	6.2	9.7	9.0	29.3	20.0	C18-20
Ano	5	0.4	0.6	0.6	8	0.5	0.8	0.8	6	0.4	0.6	0.5	-25.0	-37.5	C21
Hígado	26	1.9	2.9	2.7	32	2.1	3.2	3.0	34	2.0	3.2	2.8	0.0	-6.7	C22
Vesícula biliar	59	4.3	6.5	6.4	48	3.1	4.8	5	47	2.8	4.4	4	-8.3	-20.0	C23-24
Páncreas	21	1.5	2.3	2.3	54	3.5	5.4	5.5	41	2.4	3.8	3.7	-29.6	-32.7	C25
Fosas nasales y oído medio	0	0.0	0.0	0.0	3	0.2	0.3	0.3	2	0.1	0.2	0.1	-33.3	-66.7	C30-31
Laringe	0	0.0	0.0	0.0	1	0.1	0.1	0.1	1	0.1	0.1	0.1	0.0	0.0	C32
Pulmón	23	1.7	2.5	2.3	37	2.4	3.7	3.8	57	3.4	5.3	4.8	43.2	26.3	C33-34
Otros órganos torácicos	2	0.1	0.2	0.3	2	0.1	0.2	0.1	3	0.2	0.3	0.3	50.0	200.0	C37-38
Hueso	5	0.4	0.6	0.6	8	0.5	0.8	0.7	8	0.5	0.7	0.7	-12.5	0.0	C40-41
Melanoma de piel	31	2.3	3.4	3.3	32	2.1	3.2	3.2	45	2.7	4.2	4.0	31.3	25	C43
Otros de piel	10	0.7	1.1	1.1	10	0.6	1.0	1.1	15	0.9	1.4	1.2	40.0	9.1	C44
Mesotelioma	3	0.2	0.3	0.4	0	0.0	0.0	0.0	1	0.1	0.1	0.1			C45
Sarcoma de Kaposi	0	0.0	0.0	0.0	1	0.1	0.1	0.1	0	0.0	0.0	0.0	-100.0	-100.0	C46
Tejidos conjuntivos y blandos	15	1.1	1.7	1.6	15	1.0	1.5	1.6	11	0.7	1.0	0.9	-33.3	-43.8	C47,49
Mama	219	16.1	24.2	24.4	258	16.7	25.9	27.1	313	18.6	29.3	27.7	13.1	2.2	C50
Vulva	5	0.4	0.6	0.5	5	0.3	0.5	0.6	6	0.4	0.6	0.5	20.0	-16.7	C51
Vagina	7	0.5	0.8	0.7	0	0.0	0.0	0.0	3	0.2	0.3	0.3			C52
Cuello del útero	251	18.5	27.7	27	272	17.7	27.3	27.4	210	12.5	19.6	18.0	-28.2	-34.3	C53
Cuerpo del útero	39	2.9	4.3	4.6	45	2.9	4.5	4.7	45	2.7	4.2	4.3	-6.7	-8.5	C54
Útero NOS	4	0.3	0.4	0.4	1	0.1	0.1	0.1	4	0.2	0.4	0.4	300.0	300.0	C55
Ovario	63	4.6	7.0	7.1	70	4.5	7.0	7.1	84	5.0	7.9	7.3			C56
Placenta	2	0.1	0.2	0.2	4	0.3	0.4	0.3	0	0.0	0.0	0.0	-100.0	-100.0	C58
Riñón	13	1.0	1.4	1.4	13	0.8	1.3	1.4	15	0.9	1.4	1.5	7.7	7.1	C64
Pelvis renal	1	0.1	0.1	0.1	0	0.0	0.0	0.0	1	0.1	0.1	0.1			C65
Vejiga	18	1.3	2.0	2.0	14	0.9	1.4	1.5	20	1.2	1.9	1.7	35.7	13.3	C67
Otros órganos urinarios	0	0.0	0.0	0.0	1	0.1	0.1	0.1	1	0.1	0.1	0.1	0.0	0.0	C68
Ojo	10	0.7	1.1	1.2	7	0.5	0.7	0.7	2	0.1	0.2	0.2	-71.4	-71.4	C69
Cerebro y SNC	23	1.7	2.5	2.7	27	1.8	2.7	2.8	49	2.9	4.6	4.4	70.4	57.1	C70-72
Glándula tiroides	71	5.2	7.8	7.7	92	6.0	9.2	9.0	139	8.3	13	11.8	41.3	31.1	C73
Glándula suprarrenal	1	0.1	0.1	0.2	0	0.0	0.0	0.0	0	0.0	0.0	0.0			C74
Otras glándulas endocrinas	0	0.0	0.0	0.0	0	0.0	0.0	0.0	2	0.1	0.2	0.2			C75
Linfomas	64	4.7	7.1	6.6	71	4.6	7.1	7.3	75	4.5	7.0	6.3	-1.4	-13.7	C81-82,85,96
Mieloma múltiple	7	0.5	0.8	0.8	19	1.2	1.9	2.1	16	1.0	1.5	1.4			C90
Leucemias	35	2.6	3.9	4.2	35	2.3	3.5	3.7	45	2.7	4.2	4.1	20.0	10.8	C90-95
Síndromes mielodisplásicos	0	0.0	0.0	0.0	3	0.2	0.3	0.3	12	0.7	1.1	0.9	266.7	200.0	CIE-O-3: 998_/3
Desórdenes mieloproliferativos	0	0.0	0.0	0.0	1	0.1	0.1	0.1	8	0.5	0.9	0.8	800.0	700.0	CIE-O3: 9950/3, 996_3/3, 9975/3
Sitios NOS	48	3.5	5.3	5.6	65	4.2	6.5	6.4	98	5.8	9.2	8.8	41.5	37.5	C26,39,48,76,80
Todas las localizaciones	1,359	100.0	149.9	149.6	1,541	100.0	154.7	156.1	1,684	100.0	157.7	147.1	1.9	-5.8	C00-96
Todas las localizaciones sin piel	1,349	99.3	148.8	148.5	1,531	99.4	153.7	155.1	1,669	99.1	156.3	145.9	1.7	-5.9	C00-43,45-96

TCI: Tasa cruda de incidencia x 100,000 mujeres-año;

TAE: Tasa ajustada por edad x 100,000 mujeres-año (población mundial estándar Segi)

PC: Porcentaje de cambio

Según el sexo y periodo, en hombres se presentaron entre 1998 y 2002, 982 casos (TAE 135 casos por 100,000 hombres-año), para 2003-2007 1,174 casos (TAE 147.1 casos por 100,000 hombres-año) y en 2008-2012 1,270 casos (TAE 136.4 casos por 100,000 hombres-año). En mujeres entre 1998 y 2002 se presentaron 1,359 casos (TAE 149.6 casos por 100,000 mujeres-año), para 2003-2007: 1,541 casos (TAE 156.1 casos por 100,000 mujeres-año) y en 2008-2012: 1,684 casos (TAE 147.1 casos por 100,000 mujeres-año). El porcentaje de cambio de las tasas globales de incidencia entre los últimos dos periodos fue de 0.2 en hombres y en mujeres del 1.0%.

La tendencia de la incidencia indica que no hubo cambios significativos en las tasas de incidencia tanto en hombres como en mujeres durante el periodo analizado (*p*-valor hombres= 0.7; *p*-valor mujeres= 0.3), es decir que la incidencia fue estable. El promedio de las tasas anuales de incidencia ajustada por edad fue de 139.7 casos por 100,000 hombres-año y de 150.7 casos por 100,000 mujeres-año (Fig. 2).

**Figura 2:** Tendencia de las tasas globales de incidencia de cáncer ajustadas por edad. Hombres y mujeres de Pasto, Colombia, 1998-2012. APC=Porcentaje de cambio anual. TAE: Tasa ajustada por edad (población mundial estándar Segi) x 100,000 hombres o mujeres-año.

### Incidencia por edad

Los casos y las tasas de incidencia de cáncer aumentaron con la edad; el 55% de los casos incidentes en hombres y el 40% en mujeres se presentaron a partir de los 65 años, mientras que, el 2% de los casos en ambos géneros ocurrieron en menores de 15 años (Fig. 3). La edad promedio de diagnóstico en hombres fue de 62.3 años (DE=18.7 años) y en mujeres de 58.2 años (DE=18.1 años). 

**Figura 3:** Casos y tasas específicas por edad por 100,000 hombres o mujeres-año de incidencia de cáncer, Pasto, Colombia, 1998-2012.

### Incidencia por tipo de tumor

Los tumores más frecuentes durante los 15 años en hombres fueron de: Estómago (23.5%), próstata (18.3%), linfomas (7.6%) colon y recto (5.2%) y pulmón (4.9%). En mujeres fueron de: mama (17.3%), cuello del útero (16.0%), estómago (10.8%), tiroides (6.6%) y colon y recto (5.3%). Este comportamiento se observó cuando se analizó cada uno de los tres quinquenios que comprendió el periodo de estudio (Tablas 2 y 3).

La incidencia de los tumores de estómago en hombres disminuyó de forma significativa un 4.6% (*p*-valor= 0.0) anualmente desde una TAE de 41.5 en 1998 a 19.1 casos por 100,000 hombres-año en 2012. En mujeres, la incidencia del cáncer de cuello uterino disminuyó 8.6% (*p*-valor= 0.0) anualmente a partir de 2003 desde una TAE de 34.2 hasta 13.4 casos por 100,000 mujeres-año en 2012. La incidencia de los tumores de próstata (*p*-valor= 0.2) y mama (*p*-valor= 0.3) fue constante y el promedio de sus tasas anuales de incidencia ajustada por edad fue de 26 casos por 100,000 hombres-año y 26.4 casos por 100,000 mujeres-año, respectivamente (Fig. 4).

**Figura 4:** Tendencia de las tasas de incidencia ajustadas por edad de los tumores más frecuentes. Pasto, Colombia, hombres y mujeres 1998-2012.

## Mortalidad global 

Durante el periodo 1998-2012 en el municipio de Pasto se presentaron 4,221 muertes de cáncer (TAE 75.9 muertes por 100,000 personas-año), 1,949 en hombres (TAE 78 muertes por 100,000 hombres-año) y 2,272 muertes en mujeres (TAE 74.4 muertes por 100,000 mujeres-año (Tablas 4 y 5).

**Tabla 4:** Tasas de mortalidad por cáncer según localización, crudas y estandarizadas por edad por 100,000 hombres-año. Pasto, Colombia, 1998-2002, 2003-2007 y 2008-2012.

Site	1998-2002				2003-2007				2008-2012				PC (%) 2003-2007 and 2008-2012		ICD-10 code
	n	%	TC	TAE	n	%	TC	TAE	n	%	TC	TAE	TC	TAE	
Oral cavity	3	0.5	0.4	0.4	4	0.6	0.4	0.4	9	1.2	0.9	0.9	125.0	125.0	C00-14
Oesophagus	22	4.0	2.6	3.2	25	3.9	2.7	3.1	25	3.3	2.5	2.6	-7.4	-16.1	C15
Stomach	190	34.4	22.3	26.6	172	26.5	18.8	21.5	200	26.7	20.2	21.5	7.4	0.0	C16
Small intestine	0	0.0	0	0	2	0.3	0.2	0.2	2	0.3	0.2	0.2	0.0	0.0	C17
Colon and rectum	15	2.7	1.8	1.9	34	5.2	3.7	4.2	50	6.7	5.1	5.3	37.8	26.2	C18-20
Liver	28	5.1	3.3	4.3	48	7.4	5.2	6.4	31	4.1	3.1	3.2	-40.4	-50.0	C22
Pancreas	20	3.6	2.3	3.1	28	4.3	3.1	3.4	27	3.6	2.7	3.0	-12.9	-11.8	C25
Lung	62	11.2	7.3	9.2	61	9.4	6.7	7.3	66	8.8	6.7	7.3	0.0	0.0	C34
Skin melanoma and Other skin	9	1.6	1.1	1.3	2	0.3	0.2	0.3	11	1.5	1.1	1.1	450.0	266.7	C43-44
Breast	2	0.4	0.2	0.2	1	0.2	0.1	0.1	0	0.0	0.0	0.0	-100.0	-100.0	C50
Próstate	66	11.9	7.7	9.4	81	12.5	8.9	9.2	92	12.3	9.3	9.0	4.5	-2.2	C61
Bladder	9	1.6	1.1	1.4	7	1.1	0.8	0.7	17	2.3	1.7	1.7	112.5	142.9	C67
Lymphomas and myelomas	37	6.7	4.3	4.4	45	6.9	4.9	5.7	55	7.4	5.6	6.0	14.3	5.3	C90 ,C81-82,85,96
Leucemias	25	4.5	2.9	2.8	21	3.2	2.3	2.4	42	5.6	4.2	4.3	82.6	79.2	C90-95
Other malignant tumours	65	11.8	7.6	8.8	117	18.1	12.8	14.4	121	16.2	12.2	12.9	-4.7	-10.4	
All sites	553	100.0	64.9	76.9	648	100.0	70.8	79.4	748	100.0	75.6	79.0	6.8	-0.5	C00-96

CR: Crude rate of mortality x 100,000 males-year;

ASR: Age-standardized rates (SEGI world population standard) x 100,000 males-year;

PC: Percentage of change

**Tabla 5:** Tasas de mortalidad por cáncer según localización, crudas y estandarizadas por edad por 100,000 mujeres-año. Pasto, Colombia, 1998-2002, 2003-2007 y 2008-2012.

Localización	1998-2002				2003-2007				2008-2012				PC(%) 2003-2007 and 2008-2012		Código CIE-10
	n	%	TC	TAE	n	%	TC	TAE	n	%	TC	TAE	TC	TAE	
Cavidad bucal	2	0.3	0.2	0.2	3	0.4	0.3	0.3	7	0.7	0.7	0.6	133.3	100	C00-14
Esófago	8	1.3	0.9	1.0	11	1.6	1.1	1	13	1.3	1.2	1.1	9.1	10	C15
Estómago	131	21.8	14.5	14.1	121	17.3	12.1	12	128	13.2	12	10.9	-0.8	-9.2	C16
Intestino delgado	3	0.5	0.3	0.3	3	0.4	0.3	0.3	1	0.1	0.1	0.1	-66.7	-66.7	C17
Colon y recto	25	4.2	2.8	2.5	41	5.8	4.1	4.1	74	7.6	6.9	6.3	68.3	53.7	C18-20
Hígado	31	5.2	3.4	3.6	44	6.3	4.4	4.2	46	4.7	4.3	4.1	-2.3	-2.4	C22
Páncreas	36	6.0	4.0	4.0	52	7.4	5.2	5.3	43	4.4	4.0	3.7	-23.1	-30.2	C25
Pulmón	38	6.3	4.2	4.0	41	5.8	4.1	4.2	62	6.4	5.8	5.5	41.5	31	
Melanoma de piel y otros de piel	13	2.2	1.4	1.4	11	1.6	1.1	1.0	17	1.8	1.6	1.4	45.5	40	C43-44
Mama	69	11.5	7.6	7.6	73	10.4	7.3	7.6	115	11.9	10.8	10.4	47.9	36.8	C50
Cuello del útero	107	17.8	11.8	11.6	65	9.3	6.5	6.6	111	11.4	10.4	9.5	60	43.9	C53
Cuerpo del útero	2	0.3	0.2	0.2	14	2.0	1.4	1.5	5	0.5	0.5	0.5	-64.3	-66.7	C54
Ovario	0	0.0	0.0	0.0	30	4.3	3	3.2	56	5.8	5.2	5	73.3	56.3	C56
Vejiga	7	1.2	0.8	0.8	5	0.7	0.5	0.5	14	1.4	1.3	1.3	160	160	C67
Linfomas y mieloma	34	5.7	3.8	3.8	34	4.9	3.4	3.5	48	4.9	4.5	4.2	32.4	20	C90,81-82,85,96
Leucemias	30	5	3.3	3.3	24	3.4	2.4	2.5	41	4.2	3.8	3.5	58.3	40	C90-95
Otras neoplasias malignas	65	10.8	7.2	7.2	129	18.4	13	13.2	189	19.5	17.7	16.3	36.2	23.5	
Todos los sitios	601	100.0	66.3	65.6	701	100.0	70.4	71.2	970	100.0	90.7	84.3	28.8	18.4	C00-96

TC: Tasa cruda de mortalidad x 100,000 mujeres-año;

TAE: Tasa ajustada por edad (población mundial estándar Segi) x 100,000 mujeres-año;

PC: Porcentaje de cambio

Según el sexo y periodo, en hombres se presentaron entre 1998 y 2002, 553 muertes (TAE: 76.9 muertes por 100,000 hombres-año), para 2003-2007, 648 muertes (TAE: 79.4 muertes por 100,000 hombres-año) y en 2008-2012, 748 muertes (TAE: 79 por 100,000 muertes hombres-año). En mujeres durante 1998 y 2002 se presentaron 601 muertes casos (TAE: 65.6 muertes por 100,000 mujeres-año), para 2003-2007, 701 muertes (TAE: 71.2 muertes por 100,000 mujeres-año) y en 2008-2012, 970 muertes (TAE: 84.3 muertes por 100,000 mujeres-año).

La tendencia de la mortalidad indica que no hubo cambios significativos en las tasas de mortalidad en hombres (*p*-valor hombres= 1.0; *p*-valor mujeres= 0.0), el promedio de la tasa anual de mortalidad ajustada por edad fue de 78.5 muertes por 100,000 hombres-año. Mientras que en mujeres las tasas de mortalidad se incrementaron 2.1% anualmente de una TAE de 58.6 a 86.0 muertes por 100,000 mujeres-año entre 1998 y 2012 (Fig. 5). El promedio en los tres quinquenios de la razón entre las tasas de mortalidad-incidencia (M:I) fue de 56 muertes por cada 100 casos diagnosticados en hombres y de 49 muertes por cada 100 casos diagnosticados en mujeres.

**Figura 5:** Tendencia de las tasas globales de mortalidad por cáncer ajustadas por edad. Hombres y mujeres. Pasto, Colombia, 1998-2012.

### Mortalidad por edad

Al igual que la incidencia, la mortalidad presentó el mayor porcentaje de casos a partir de los 65 años, tanto en hombres (63.9%) como en mujeres (55.2%). (Fig. 6) La edad promedio de muerte para hombres fue estimada en 66 años (DE=17.8 años) y para mujeres en 63.7 años (DE=17.1). 

**Figura 6:** Muertes y tasas de mortalidad por cáncer específica por edad por 100,000 hombres o mujeres-año. Pasto, Colombia, 1998-2012. TEE: Tasa especifica por edad x 100,000 hombres o mujeres-año

### Mortalidad por tipo de tumor

Durante 1998-2012, las principales causas de mortalidad por cáncer en hombres fueron los tumores de: Estómago (28.8%), próstata (12.3%) pulmón (9.7%), linfomas y mielomas (7%) y hígado (5.5%). En mujeres fueron los tumores de: Estómago (16.7%), cuello del útero (12.5%), mama (11.3%), pulmón (6.2%), colon y recto (6.2%). Este comportamiento se observó durante los tres quinquenios que comprende el periodo de estudio (Tablas 4 y5).

La mortalidad causada por los tumores de estómago en hombres disminuyó de forma significativa (*p*-valor= 0.0) 2.18% anualmente desde una TAE de 24.5 en 1998 a 19.9 muertes por 100,000 hombres-año en 2012. En mujeres, la mortalidad por cáncer de mama aumentó (*p*-valor= 0.0) 3% anualmente a partir de una TAE de 6.3 en 1998 a 10.8 muertes por 100,000 mujeres-año en 2012. La mortalidad por tumores de próstata (*p*-valor= 0.9) estomago en mujeres (*p*-valor= 0.4) y cuello uterino (*p*-valor= 0.4) fue constante y el promedio de sus tasas anuales de mortalidad ajustada por edad fue de 9.3 muertes por 100,000 hombres-año, 12.3 muertes por 100,000 mujeres-año y 9.2 muertes por 100,000 mujeres-año, respectivamente (Fig. 7).

**Figura 7:** Tendencia de las tasas de mortalidad ajustadas por edad por los tumores más frecuentes. Pasto, hombres y mujeres 1998-2012. TAE: Tasa ajustada por edad x 100,000 hombres o mujeres-año

## Discusión

La caracterización poblacional sobre la incidencia y mortalidad por cáncer que realizó este estudio hace parte de la iniciativa para implementar programas para su control que promueven a nivel global la Organización de las naciones unidas, la Organización Mundial de la Salud (OMS) la agencia especializada en cáncer la IARC y a nivel nacional el Ministerio de Salud y Protección Social y el Instituto Nacional de Cancerología de Colombia ^10^^,^^17^. Estas organizaciones consideran que la implementación de cualquier programa para el control de esta enfermedad y sus desenlaces debe estar sustentada en evidencia científica y los definen como: *“abordajes desde la salud pública, diseñados para contribuir en la reducción de la morbi-mortalidad por cáncer, mejorar la calidad de vida de los pacientes con esta afección a través de la imple mentación sistemática y equitativa de estrategias de prevención, diagnóstico temprano, tratamiento y paliación basadas en la evidencia, para el óptimo uso de los recursos disponibles; priorizando en aquellas poblaciones vulnerables con mayor carga de cáncer e interviniendo en aquellos tumores que producen mayor incidencia y mortalidad”* ^3^^,^^18^^-^^20^. 

A nivel regional, gracias a los avances del Registro de Cáncer de Cali para el reporte sistemático de datos sobre incidencia, mortalidad y supervivencia a cáncer, la Unión para el Control Internacional del Cáncer (UICC), seleccionó a la ciudad de Cali como una de las primeras ciudades del mundo para implementar la iniciativa C/Can 2025: Desafío de ciudades contra el cáncer que consiste en involucrar a todos los actores interesados de cada ciudad, en el diseño, planificación e implementación de soluciones para el cuidado del cáncer. Los datos de la caracterización de la morbi-mortalidad por cáncer que provee el Registro de cáncer de Cali desde hace cinco décadas serán la única fuente de evidencia para la evaluación de los efectos de la implementación de ésta estrategia ^21^. Siguiendo el modelo de Cali, se pretende que los resultados que se presentan en este estudio constituyan la línea de base sobre la situación del cáncer en el municipio de Pasto para evaluar los efectos de la implementación del *Plan Nacional para el Control del Cáncer* que se planteó en Colombia en el 2010 ^9^*.*

El Registro Poblacional de Cáncer en el Municipio de Pasto inició la recolección de datos desde 1998 sobre la magnitud del cáncer con el fin de realizar el seguimiento y vigilancia de la evolución de la morbi-mortalidad, servir como insumo en la toma de decisiones a nivel institucional, regional y nacional y fomentar la producción de conocimiento con el fin de disminuir y controlar de la enfermedad ^22^^,^^23^. 

En general, se considera que la tasa de incidencia global para el Municipio de Pasto (TAE:139.1 casos por 100,000 hombres-año y TAE:150.3 casos por 100,000 mujeres-año) y en otras regiones cercanas geográficamente son bajas; Cali-Colombia (TAE: 205 casos por 100,000 hombres-año y TAE:186 casos por 100,000 mujeres-año), Manizales-Colombia (TAE:156.0 casos por 100,000 hombres-año y TAE:165.0 casos por 100,000 mujeres-año), Bucaramanga-Colombia (TAE: 154 casos por 100,000 hombres-año y TAE_s_: 157.0 casos por 100,000 mujeres-año), Quito-Ecuador (TAE:193 casos por 100,000 hombres-año y TAE:199.0 casos por 100,000 mujeres-año), Manabi-Ecuador (TAE:89.0 casos por 100,000 hombres-año y TAE=102 casos por 100,000 mujeres-año) ^24^. Los resultados observados son similares en otros países Latinoamericanos y contrarios a los reportados por la mayoría de registros norteamericanos y oceánicos ^24^.

El comportamiento mundial de las tasas globales de incidencia de las poblaciones se puede asociar a estilos de vida, capacidad diagnóstica en el sistema de salud, pero principalmente a la transición demográfica y epidemiológica; en poblaciones de Norteamérica, Oceanía, Europa y Asia con un marcado envejecimiento poblacional, se incrementa el riesgo de enfermedades crónicas especialmente aquellas de presentación tardía como el cáncer, en contraste con aquellas poblaciones con una estructura poblacional más joven como en Latinoamérica y África, que tienen mayores problemas de salud pública relacionados con enfermedades trasmisibles ^25^^,^^26^.

Al contrastar las tasas de incidencia con las tasas de mortalidad, para establecer la razón mortalidad-incidencia (M:I) se puede observar que, si bien la mayoría de registros norteamericanos tienen tasas más altas de incidencia tanto en hombres como en mujeres, la razón mortalidad e incidencia alcanzó los valores más bajos (M:I =30-40), lo cual quiere decir que por cada 100 casos que se diagnosticaron se presentaron entre 30 y 40 muertes por cáncer. Por otra parte, en los países Latinoamericanos la razón M:I osciló entre 60 y 80, es decir que por cada 100 casos incidente se presentaron aproximadamente 80 muertes. Este un indicador indirecto de la calidad del sistema de salud en relación a las pruebas diagnósticas para la identificación de nuevos casos de cáncer y a los servicios de tratamiento oncológico para evitar las muertes ^7^.

Cuando se compara las tendencias de la incidencia y mortalidad reportada por otros registros que han publicado al menos 15 años de forma consecutiva en CI5, se observa que la incidencia de los tumores de estómago disminuyó de forma significativa en la mayoría de poblaciones, primordialmente en aquellas de países europeos donde el PCA reportado oscila entre -5.1% y -3.1%, con menor disminución en países norteamericanos, oceánicos y algunos europeos, el PCA varía entre -2.9% y -2.3% y con la más baja disminución las poblaciones Latinoamericanas con un PCA que oscila entre -2.2% y -0.9%. Únicamente el registro de kyadondo county-Uganda y Goiania- Brasil, han reportado aumento de la incidencia del cáncer de estómago donde el PCA 2.1% y 0.1% respectivamente. En el Municipio de Pasto la tendencia de la incidencia y mortalidad por cáncer de estómago ha logrado una disminución significativa, este comportamiento lo explica el estudio sobre tendencia de la incidencia y mortalidad por cáncer de estómago en Cali que refiere que probablemente esté relacionado con la disminución gradual de las tasas de prevalencia de la infección por *Helicobacter pylori*, la mejora de los hábitos vida y detección temprana en la población^27^^,^^28^, sin embargo, para el caso del Municipio de Pasto se sugiere estudiar a profundidad las causas de este comportamiento. 

La tendencia de la incidencia de los tumores de próstata en el mundo ha aumentado de forma significativa en la mayoría de poblaciones; de forma acelerada en poblaciones latinoamericanas, algunas europeas, asiáticas y oceánicas con un PCA que oscilan entre 5.2% y 11.0%, ha elevado levemente en algunos países europeos, asiáticos y oceánicos con un PCA que fluctúa entre 3.4% y 3.9%, y en países africanos, norteamericanos y algunos europeos ha aumentado muy poco, con un PCA que oscila entre 3.3% y 1.5%. Solo poblaciones de la India (3 registros) han reportado que la tendencia de la incidencia del cáncer de próstata ha disminuido levemente con un PCA de 0.2%. Para el Municipio de Pasto las tasas de incidencia y mortalidad a lo largo del tiempo se han manteni do constantes sin evidenciar un cambio estadísticamente significativo. El análisis de éste comportamiento debe ser objeto de estudio para establecer los factores relacionados con el diagnóstico temprano y tratamiento.

En mujeres la tendencia de la incidencia de cáncer de mama esta aumentado de forma global y se acentúa en poblaciones de registros africanos y algunos europeos y asiáticos con un PCA que oscila entre 5.3% y 2.2%, en poblaciones latinoamericanas, oceánicas y algunas europeas ha aumentado medianamente con un PCA que varía entre 2.1% y 1.3%, y muy poco en poblaciones norteamericanas y algunos asiáticos con un PCA que oscila entre 0.3% y 1.2%. Este comportamiento probablemente causado por el aumento de la obesidad inactividad física, cambios en los hábitos reproductivos y otros comportamentales ^29^. La tendencia de la mortalidad por cáncer de mama varía entre regiones: poblaciones de países europeos y norteamericanos muestran tendencias decrecientes, contrario a lo presentado en las poblaciones de países suramericanos. En Pasto la tasa de incidencia se mantiene constante y la mortalidad tiene una tendencia al aumento similar a otras poblaciones de países suramericanos ^30^. 

La disminución de la tasas de incidencia del cáncer de cuello uterino a nivel mundial es muy evidente, con un descenso mayor en poblaciones Latinoamericanas, oceánicas y algunas europeas con un PCA que oscila entre -2.7% y -8.6%, una disminución media en poblaciones norteamericanas, algunas europeas y asiáticas con un PCA que fluctúan entre -1.4% y -2.6% y con una disminución reducida en algunas poblaciones europeas y asiáticas con un PCA que oscila entre -0.3% y -1.1%, comportamiento contrario a la reportada por poblaciones africanas donde la tendencia de la incidencia aumenta con un PCA de 3,9%. En Pasto la tendencia de la incidencia de cáncer de cuello uterino ha bajado sin embargo la tendencia de la mortalidad se mantiene constante, resultados que reflejan dificultades en la detección temprana y tratamiento oportuno. 

Para el Municipio de Pasto el análisis de la incidencia, mortalidad y el comportamiento de la tendencia de los tipos de cáncer de mayor ocurrencia se convierte en una base para la evaluación del impacto de las medidas de prevención, tratamiento, implementación de nuevas tecnologías e investigaciones que promuevan acciones para controlar el impacto de la enfermedad en la población.
